# Me6TREN targets β-catenin signaling to stimulate intestinal stem cell regeneration after radiation

**DOI:** 10.7150/thno.46415

**Published:** 2020-08-13

**Authors:** Sihan Wang, Yi Han, Jing Zhang, Shu Yang, Zeng Fan, Feiling Song, Lijuan He, Wen Yue, Yanhua Li, Xuetao Pei

**Affiliations:** 1 Stem Cells and Regenerative Medicine Lab, Institute of Health Service and Transfusion Medicine, Beijing 100850, China.; 2 Experimental Hematology and Biochemistry Lab, Beijing Institute of Radiation Medicine, Beijing 100850, China.; 3 South China Research Center for Stem Cell & Regenerative Medicine, SCIB, Guangzhou 510005, China.

**Keywords:** Me6, Intestinal stem cell, Regeneration, Organoid, β-catenin

## Abstract

**Background:** Acute gastrointestinal syndrome (AGS) is one of the most severe clinical manifestations after exposure to high doses of radiation, and is life-threatening in radiological emergency scenarios. However, an unmet challenge is lacking of an FDA-approved drug that can ameliorate the damage of radiation-exposed intestinal tissues and accelerate the regeneration of injured epithelia. In this study, we investigated whether the small molecule Me6TREN (Me6) can regulate intestinal stem cell (ISC) proliferation and promote crypt regeneration after irradiation.

**Methods:** Lethally irradiated mice were administered with Me6 or PBS to study the survival rate, and sections of their small intestine were subjected to immunostaining to evaluate epithelial regeneration. An intestinal organoid culture system was employed to detect the role of Me6 in organoid growth and ISC proliferation. We further investigated the key signaling pathways associated with Me6 using microarray, western blotting, and RNA interference techniques.

**Results:** We identified the small molecule Me6 as a potent intestinal radiation countermeasure. Systemic administration of Me6 significantly improved ISC and crypt cell regeneration and enhanced the survival of mice after high doses of radiation. Using an *in vitro* intestinal organoid culture system, we found that Me6 not only induced ISC proliferation but also increased the budding rate of intestinal organoids under unirradiated and irradiated conditions. Me6 remarkably activated the expression of ISC-associated and proliferation-promoting genes, such as *Ascl2*,* Lgr5*,* Myc,* and *CyclinD1*. Mechanistically, Me6 strongly stimulated the phosphorylation of β-catenin at the S552 site and increased the transcriptional activity of β-catenin, a key signaling pathway for ISC self-renewal and proliferation. This is further evidenced by the fact that knockdown of β-catenin abolished the effect of Me6 on intestinal organoid growth *in vitro* and crypt regeneration in irradiated mice.

**Conclusion:** The small molecule Me6TREN induced ISC proliferation, enhanced intestinal organoid growth *in vitro*, and promoted intestinal tissue regeneration after radiation injury by activating β-catenin signaling.

## Introduction

The gastrointestinal tract is one of the most radiosensitive organs, as shown by easily getting injured and resulting in acute gastrointestinal syndrome (AGS) after a high dose of ionizing radiation. AGS can lead to severe disorders, including lethality, in animals and humans, and it is also a major dose-limiting complication in patients receiving abdominal or pelvic radiotherapy 1, 2. It is an unmet requirement to find ways to protect intestinal tissue, accelerate epithelial regeneration, and prevent mortality of individuals from radiation-induced damage. Accumulating evidence has suggested that a variety of agents such as blockers of oxygen consumption, free radical scavengers, death pathway modulators, growth factors, and CDK4/6 inhibitors have protective roles in animal models with multiple types of intestinal radiation damage 3-5. Despite significant scientific advances towards the development of a safe and effective radiation countermeasure for AGS, no drug has been approved to be used in the clinic by the US FDA. There is an urgent need to develop radiation countermeasure agents, especially chemical compounds, to accelerate regeneration of injured intestinal tissue and decrease life-threatening sequelae.

Intestinal stem cells (ISCs) play a critical role in the maintenance of cell populations of the intestinal epithelium and in driving intestinal tissue regeneration after injury. Crypt-based columnar cells (CBCs) with Lgr5 marker have been shown to be active stem cells with self-renewal and multipotential characteristics 6, 7. The cells located at the crypt base +4 position were shown to function as reserve ISCs with slow cycling features, which can convert into Lgr5^+^ CBCs and support regeneration under stressful circumstances 8. The fate of ISCs is regulated by complex extracellular signaling pathways in the crypt niche, including Wnt, Notch, and BMP signaling 9-12. Among these pathways, Wnt-driven signaling is indispensable for crypt proliferation and regulation of ISC identity 13. Key genes such as *Ascl2* and *Sox9* are downstream targets of the Wnt/β-catenin signaling pathway and are crucial in regulating the ISC state 14, 15. Therefore, pharmaceutical interventions, especially using small molecules to regulate these signaling pathways, might provide an effective therapeutic method for promoting intestinal tissue regeneration and mitigating radiation-induced AGS in radiation-exposed individuals.

Me6TREN (Me6: Tris [2-(dimethylamino)ethyl]amine) is an alkaloid analogue that contains multiple hydrogen-bonding acceptor sites. It is frequently used as a ligand for the synthesis of functional water-soluble polymers 16. Thus far, less is known about the biological activity of Me6. We recently reported that Me6 is a potent and effective agent for hematopoietic stem and progenitor cell mobilization 17. Me6 was shown to enhance ischemic tissue angiogenesis and promote ischemic limb repair 18. Therefore, we wanted to determine whether Me6 could accelerate radiation-injured tissue regeneration. In this study, we found that Me6 administration significantly enhanced intestinal epithelium repair using histopathological analysis of intestinal tissue seven days after irradiation. Utilizing a high-dose total body or abdominal irradiation model and an *in vitro* intestinal organoid culture system, we characterized the role of Me6 in crypt regeneration following radiation injury, and deciphered the molecular mechanism by which Me6 induced ISC proliferation and promoted new crypt formation. Our findings showed that Me6 could be a potent and effective therapeutic agent for crypt regeneration and intestinal tissue repair after irradiation.

## Materials and Methods

### Mice and radiation

Mice received whole-body irradiation at doses ranging from 8 Gy to 14 Gy using a ^60^Co irradiator (Beijing Institute of Radiation Medicine, Beijing). Mice received abdomen X-ray irradiation using a RS-2000 Pro Biological System. The survival time of mouse after irradiation was observed for 30 days.

### Me6 treatment schedule

The small molecule compound Me6 (Sigma) was dissolved in PBS and subcutaneously injected to the mice at 2.5 mg/kg on days 0, 3, 6, 9, and 12 after irradiation. The first dose of Me6 was administered immediately in mice within 1 h after radiation exposure. Control animals were injected with the same volume of PBS.

### Immunohistochemistry assays

Paraffin sections of the jejunum were rehydrated for 5 min in 100%, 90% and 75% ethanol, and then were subsequently rinsed in 0.1% Triton X-100 in phosphate-buffered saline (PBS). The samples were incubated with primary antibodies in 1% bovine serum albumin overnight at 4°C. The samples were then washed in PBS, incubated with the secondary antibodies for 60 min at room temperature, washed and mounted with NovaRed or SG (Vector Labs, Burlingame, CA). The positive cells in each crypt were determined by counting 30 intact crypts and reported as the mean ± SD. Three or more mice were used in each group. The antibodies used were as follows: anti-Lgr5 (R&D, MAB8240), anti-β-catenin (CST, 8480S), anti-PH3 (CST, 53348), anti-CyclinD1 (CST, 2978), anti-BrdU (CST, 5292), anti-Ki67 (CST, 9129), anti-p-AKT (CST, 4060), anti-p-ERK1/2 (CST, 4370), anti-Muc2 (Gene Tex, GTX100664), anti-Chga (Gene Tex, GTX113165), anti-Lysozyme (Abcam, ab108508).

### Crypt isolation and organoid culture

Mouse small intestines were cut open longitudinally and flushed with PBS. The villi were carefully removed by gentle scraping and the remaining tissue was washed with cold PBS for approximately 10 times. The tissue was cut into 2-3 mm pieces, and the tissue fragments were incubated in 2.5 mM EDTA/PBS without Ca^2+^/Mg^2+^ at 4°C for 30 min on ice. After incubation, the tissue fragments were vigorously shaken and were pelleted at 300 g with cold PBS approximately 10 times. Approximately 200 isolated crypts were embedded in Matrigel and cultured in crypt culture medium (IntestiCult™ Organoid Growth Medium, STEM CELL, 06005). The medium was changed every 2 days. The organoid formation was analyzed 7 days after plating. Three wells of a 24-well plate were used for counting the number of organoids. At least 50 organoids were used for counting the number of buds, and at least 50 organoids were used for measuring the area of organoids by Image J.

### TOP/FOP reporter assay

Every 6 × 10^5^ IEC-6 cells were transfected with a total of 1 μg Topflash (Addgene) or negative control Fopflash (Addgene) and 0.1μg of a renilla cocktail using the Neon Transfection System (Life Technologies) according to manufacturer's instructions. The conditions used were 1350 V, 30 ms, and 2-time pulses. A total of 4 × 10^4^ transfected IEC-6 cells were cultured in the medium with or without Me6 in a 24-well plate. After 48 h, IEC-6 cells were collected and treated with a Dual-Glo Luciferase Assay System (Promega, E2920) according to the manufacturer's instructions. The luciferase activity was detected by SpectraMax of Molecular Devices.

### Statistics

Data are presented as the mean ± SD. Survival was analyzed by the log-rank test. Paired *t* test was used for determination of statistical significance between two groups; one-way or two-way analysis of variance was used to compare means among three or more independent groups. *P* values less than 0.05 were considered statistically significant.

## Results

### Me6 improves the survival rate of lethally irradiated mice and enhances the repair of intestinal tissue after radiation exposure

**Figure 1:**
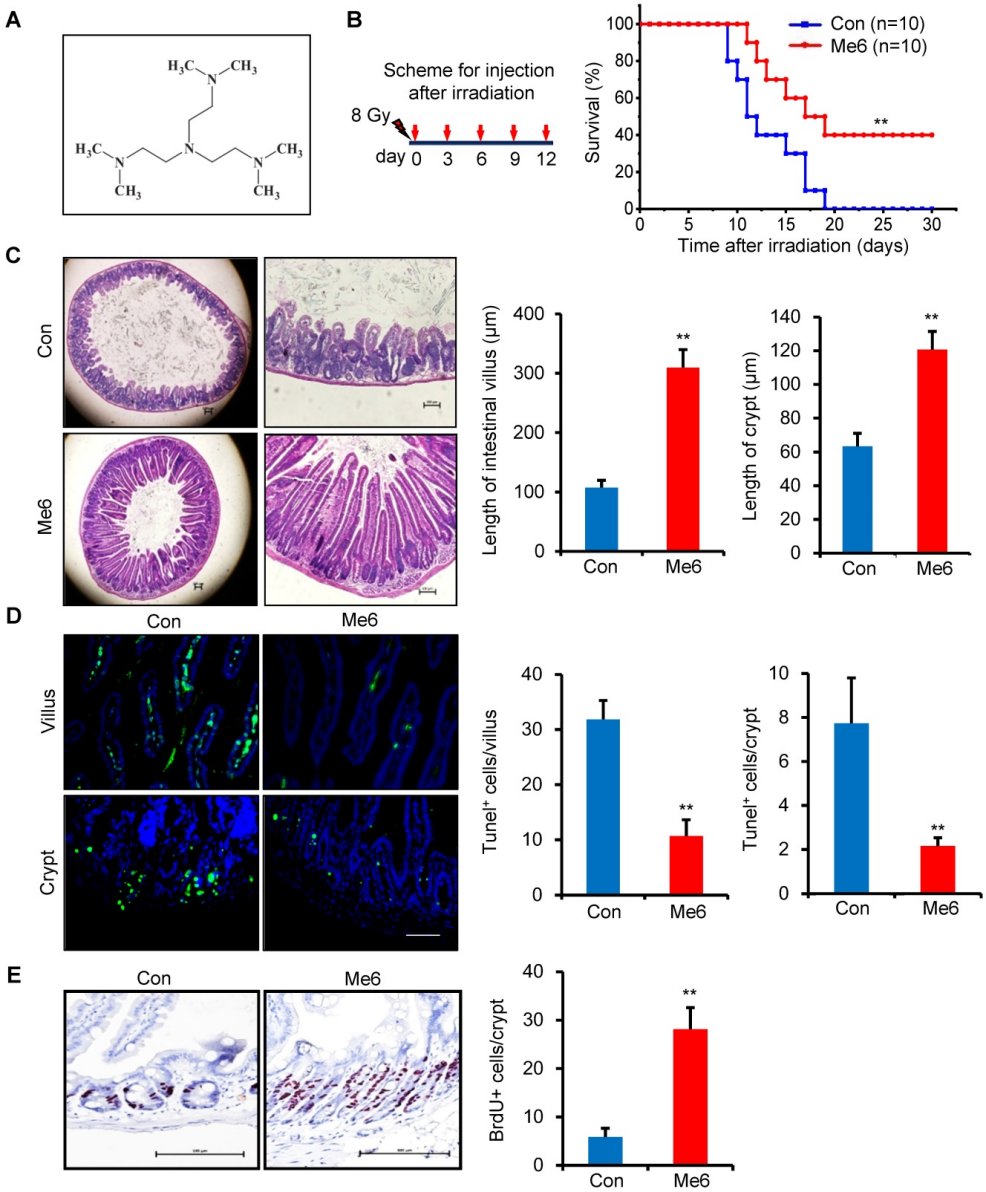
** Me6 improved the survival rate of irradiated mice and enhanced the intestinal tissue repair from radiation.** (**A**) Chemical structure of Me6. (**B**) Survival curves of Control (PBS) - or Me6-treated mice after 8 Gy whole body irradiation (WBI). Me6 at 2.5 mg/kg or PBS were administered on the indicated days after irradiation (left) (**p < 0.01). Number of animals per group is shown in parentheses. Control: Con. (**C**) Representative HE-stained sections and the quantification of the villus and crypt length of small intestine on day 7 after 8 Gy WBI and different treatments (**p < 0.01, Scale bar = 100 µm). (**D**) Representative Tunel-stained images and the quantification of the Tunel^+^ cells in the villus and crypt of the small intestine on day 7 after 8 Gy WBI and different treatments (**p < 0.01, Scale bar = 100 µm). Three mice were used in each group. (**E**) Representative BrdU-stained sections and the quantification of BrdU^+^ cells in the crypt of the small intestine after 8 Gy WBI and different treatments (**p < 0.01, Scale bar = 100 µm). Three mice were used in each group.

We initially used an end-point measurement method, that is, the survival rate of mice following whole body irradiation (WBI), to evaluate the beneficial effects of Me6 on radiation-induced tissue damage. We first chose the dose of Me6 at 5 mg/kg to treat the irradiated mice at the indicated time schedules (i.e., every one, two or three days for 12 days after radiation; Figure S1A). We found that the mice treated with Me6 on days 0, 3, 6, 9, and 12 after 8 Gy radiation had better survival than those treated with PBS or Me6 at other schedules (Figure 1A, Figure S1A). To further determine the dose-response relationship of Me6 treatment, we administered the mice with Me6 at different doses (0, 1.25, 2.5, 5, and 10 mg/kg) on days 0, 3, 6, 9, and 12 following 8 Gy radiation. The 2.5 mg/kg Me6 was observed to be the optimal dose and resulted in the highest survival rate of the mice following 8 Gy WBI (40%, Figure S1B and Figure 1B). By contrast, irradiated mice that received PBS injections died within 19 days (Figure 1B). Consistently, Me6 administration also decreased post-irradiation lethality in BALB/c mice (*p* < 0.05, Figure S2), indicating that Me6 might effectively mitigate radiation injury. Seven days after irradiation and Me6 treatment, multiple organs were removed and analyzed for histopathologic changes. Me6 administration induced better tissue morphology in the bone marrow, liver, and pulmonary tissue after irradiation (Figure S3). Notably, histological analysis of the small intestine showed that Me6 administration significantly increased intestinal villus and crypt length compared to the control group (Figure 1C). The number of apoptotic cells in the villi and crypts was markedly decreased following Me6 administration (Figure 1D). To obtain insight into the proliferative status of the intestinal crypt upon irradiation and Me6 treatment, we performed a 12 h bromodeoxyuridine (BrdU) tracer experiment. We found a 3.8-fold increase in BrdU^+^ cells in the crypts of Me6-treated mice compared to the controls on day 7 after irradiation (Figure 1E). These data suggest that Me6 improves the survival of lethally irradiated mice, promotes crypt cell proliferation, and repairs the intestinal epithelium.

### Me6 promotes intestinal stem cell proliferation and crypt regeneration after 14 Gy irradiation

**Figure 2:**
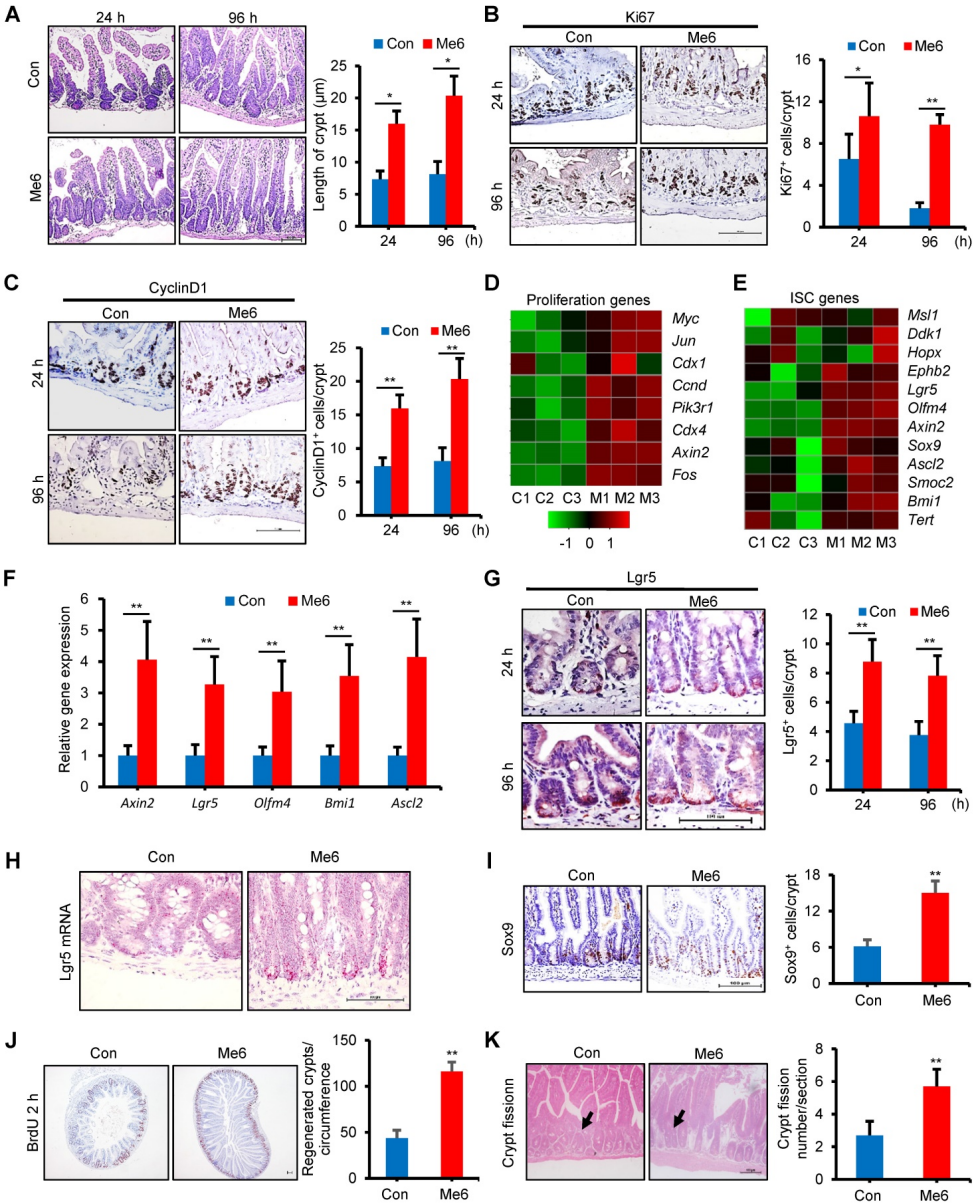
** Me6 promoted intestinal stem cell proliferation and crypt regeneration post irradiation.** (**A**) Representative HE-stained sections and the quantification of the villus and crypt length of small intestine at 24 and 96 h after 14 Gy WBI and different treatments (*p < 0.05; Scale bar = 100 µm). (**B-C**) Proliferation of the crypts of small intestine at 24 and 96 h after 14 Gy WBI and different treatments, as assessed by Ki67 (B) and CyclinD1 (C) staining, respectively (*p < 0.05, **p < 0.01; Scale bar = 100 µm). Three mice were used in each group. (**D-E**) Heat map showing the proliferation (D) and ISC-related genes (E) that are upregulated in the intestinal crypts from Me6-treated mice at 96 h after 14 Gy WBI. Microarray was performed using mRNA isolated from the intestinal crypts. (**F**) qPCR detection for ISC-related gene expression in the intestinal crypts at 96 h after 14 Gy WBI and different treatments (**p < 0.01). (**G**) Representative Lgr5-stained intestinal sections and the Lgr5^+^ cell numbers in the crypts at 24 and 96 h after 14 Gy WBI and different treatments (**p < 0.01; Scale bar = 100 µm). Three mice were used in each group. (**H**) Representative images of in situ hybridization for Lgr5 mRNA in the intestinal crypts at 96 h after 14 Gy WBI and different treatments. (**I**) Representative Sox9-stained sections and the quantification of Sox9^+^ cells in the crypts of the small intestine after 14 Gy WBI and different treatments (**p < 0.01; Scale bar = 100 µm). Three mice were used in each group. (**J**) The quantification of regenerated crypts at 96 h after 14 Gy WBI and different treatments, as indicated by BrdU incorporation for 2 h (**p < 0.01; Scale bar = 100 µm). Three mice were used in each group. (**K**) The number of crypt fission per filed on murine jejunum sections. Six mice were used in each group (**p < 0.01).

Acute and lethal intestinal crypt injury was triggered upon exposure to high doses of radiation (>12 Gy) 19-21. To further evaluate the beneficial effect of Me6 on intestinal crypt regeneration and the survival of mice, Me6 was injected into C57BL/6 mice at 2.5 mg/kg on days 0, 3, 6, 9, and 12 after receiving WBI of 14 Gy. Small intestinal tissue sections were prepared at 24 h and 96 h after irradiation, which were key time points for the detection of ISC inactivation, activation, and peak regeneration. Me6 treatment significantly improved crypt-villus architecture at 24 and 96 h after irradiation (Figure 2A). The crypt length in the Me6 group was remarkably increased by 1.8-fold at 96 h after irradiation compared to the control group (Figure 2A). Immunohistochemical staining results demonstrated significant increases in the positive rate of Ki67 and CyclinD1 during the observation period in the crypt of Me6-treated mice, as shown by the brown staining of the small intestinal tissue sections (Figure 2B-C). Me6 treatment caused 4.4-fold and 1.5-fold increases in Ki67^+^ and CyclinD1^+^ cell numbers, respectively, compared to the control group at 96 h post-irradiation (Figure 2B-C). We then performed a microarray analysis on freshly isolated crypts harvested at 96 h after irradiation with or without Me6 treatment. The data showed that several cell proliferation-promoting genes were upregulated in the Me6 treatment group (Figure 2D). We also wanted to determine whether Me6 regulated ISC gene expression *in vivo* after irradiation. Both microarray analysis and quantitative polymerase chain reaction (qPCR) results demonstrated that ISC-associated genes such as *Lgr5, Olfm4, Axin2, Ascl2,* and* Bmi1* were significantly upregulated by Me6 treatment (Figure 2E-F). Using an anti-Lgr5 antibody, we found that Me6 administration significantly enhanced the percentage of Lgr5^+^ ISCs at 24 and 96 h after irradiation compared with the control group (Figure 2G). Using an *in situ* hybridization assay, we further found that Me6 administration significantly improved the expression of Lgr5 mRNA in the intestinal crypts (Figure 2H). Consistently, Me6 treatment increased the number of Sox9^+^ cells by about 1.5-fold in the crypt (Figure 2I). Me6 administration promoted a 1.7-fold increase in the number of regenerated crypts at 96 h post-irradiation (Figure 2J). We also found that Me6 treatment led to a significant increase in crypt fission at 96 h after irradiation (Figure 2K).

**Figure 3:**
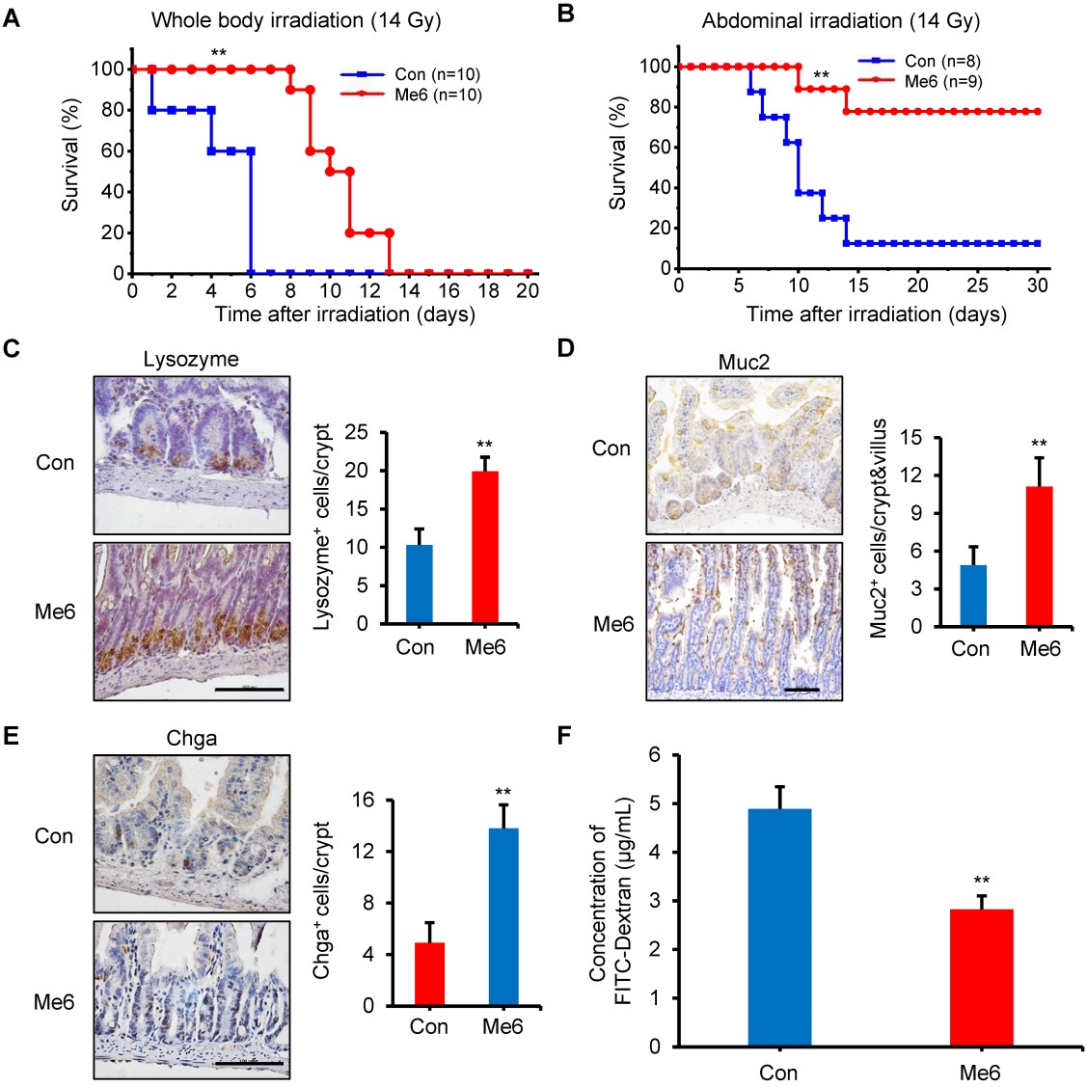
** Me6 increased the survival rate and enhanced the integrity of intestinal epithelium of mice after 14 Gy WBI.** (**A**) Survival curves of PBS (Control)- or Me6-treated mice after 14 Gy WBI. The number in parentheses indicates the number of animals per group. Me6 or PBS was administered to the mice according to the time schedule shown in Figure 1B (**p < 0.01). (**B**) Survival curves of PBS- or Me6-treated mice after 14 Gy abdominal irradiation. The number in parentheses indicates the number of animals per group (**p < 0.01). (**C-E**) Representative Lysozyme-, Muc2- and Chga-stained images and the quantification of the positive cells in the small intestine sections at 96 h after 14 Gy WBI and different treatments (**p < 0.01; Scale bar = 100 µm). Three mice were used in each group. (**F**) The FITC-Dextran levels in the serum of mice at 96 h after 14 Gy WBI and treatment with Me6 or PBS (**p < 0.01).

The survival rate experiments reflect the degree of intestinal injury and the effect of repair after a high dose of radiation. The results showed that exposure to 14 Gy WBI caused death in all C57BL/6 mice in the control group within 6 days, with a median survival time of 5 days (Figure 3A). Me6 administration significantly delayed the death of the mice by 7 days (median survival time of 10 days; P < 0.01) (Figure 3A). An abdominal irradiation (AIR) model was used to evaluate the beneficial effect of Me6 on intestinal crypt regeneration. Notably, Me6 administration significantly improved the survival rate of mice after abdominal irradiation of 14 Gy. Eighty percent of the Me6-treated mice survived compared with 15% of control mice (Figure 3B). The intestinal crypt consistently showed a better extent of repair and increased incorporation of BrdU in the Me6 treatment group seven days after irradiation (Figure S4A-C). To further assess the beneficial role of Me6 in the repair of intestinal epithelium after irradiation, we analyzed several types of intestinal epithelial cells, including paneth, goblet, and enteroendocrine cells at 96 h after 14 Gy WBI and different treatments. The results showed significant increases in the positive rate of Muc2 for goblet cells, Lysozome for paneth cells and Chromogranin A (Chga) for enteroendocrine cells on the intestinal sections of Me6-treated mice compared to the control group (Figure 3C-E), indicating that Me6 treatment enhanced the differentiation capacity of ISCs in the intestinal crypts of mice. We then employed the FITC-Dextran assay to evaluate the effect of Me6 on the intestinal permeability of mice after irradiation. Me6 administration significantly reduced FITC-Dextran levels in the serum of mice at 96 h after 14 Gy radiation (Figure 3F), indicating that Me6 administration decreased intestinal epithelial permeability and prevented gut leakiness to a greater extent in mice after irradiation. Taken together, these data indicate that Me6 administration promotes ISC proliferation and crypt regeneration, enhances the integrity of the epithelial structure of the intestine, and improves the survival rate of mice after administration of a high dose of radiation.

### Me6 promotes ISC proliferation and intestinal organoid expansion *in vitro*

**Figure 4:**
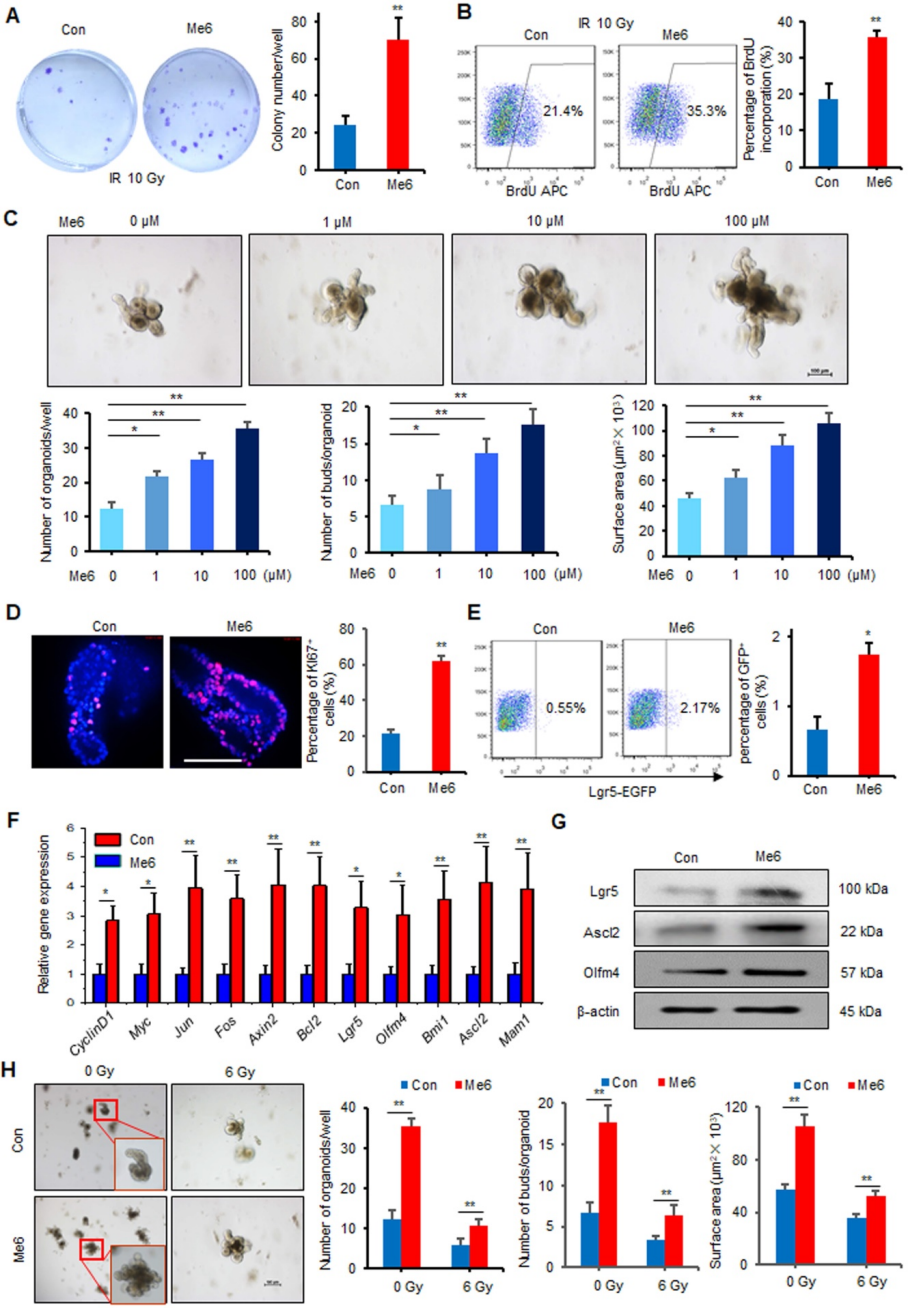
** Me6 promoted intestinal organoid expansion and ISC proliferation *in vitro*.** (**A**) Representative colony staining image and the colony numbers formed by IEC-6 cells after 10 Gy irradiation and the addition of PBS or Me6 (100 µM) to the culture medium (**p < 0.01). (**B**) The percentage analysis of BrdU incorporation for 48 h in IEC-6 cells after 10 Gy irradiation with or without the addition of Me6 (100 µM) to the medium (**p < 0.01). (**C**) Representative phase contrast microscopic images of intestinal organoids cultured with Me6 at different concentrations and the quantification of the organoid numbers per well, the bud numbers and the surface areas per organoid (*p < 0.05, **p < 0.01, Scale bar = 100 µm). (**D**) Immunostaining analysis for Ki67 in organoids treated with or without Me6 (**p < 0.01, Scale bar = 50 µm). (**E**) Representative dot plots and percentages of GFP^+^ cells in the intestinal organoids with or without Me6 (100 µM) treatment for 4 days. The intestinal crypts were isolated from Lgr5-EGFP-IRES-creERT2 mice (*p < 0.05). (**F**) qPCR for proliferation- and ISC-related gene expression in the cultured organoids with or without Me6 (100 µM) supplementation of the medium for 7 days (*p < 0.05, **p < 0.01). (**G**) Western blotting detection of ISC-related protein expression in the cultured organoids with or without Me6 (100 µM) supplementation of the medium for 7 days. (**H**) The morphology and quantification analysis of the small intestine organoids, including the numbers of organoids per well, the bud numbers and the surface areas per organoid at day 7 after irradiation (**p < 0.01). The intestinal organoids received 0 or 6 Gy irradiation and then were cultured in the standard organoid medium with or without Me6 (100 µM) supplementation.

Intestinal stem cells in the crypts play essential roles in the maintenance of intestinal homeostasis and promote intestinal regeneration after injury. To determine whether Me6 could directly regulate the proliferation of intestinal crypt cells, we first tested the effect of Me6 on cultured IEC-6 cells, a rat crypt epithelial cell line. By employing colony-forming assays, we found that Me6 treatment caused a 1.8-fold increase in the number of colonies formed by IEC-6 cells after 10 Gy irradiation (Figure 4A). The BrdU incorporation percentage of Me6-treated cells was 0.8-fold increase than that of the controls (Figure 4B). To further test the efficacy of Me6 on *in vitro* ISC proliferation, freshly isolated small intestine crypts of normal mice were cultured in standard organoid media with or without Me6 addition. Time-lapse imaging data showed that the intestinal organoids were larger in size and showed more budding after Me6 supplementation during the observation period (Video S1, Figure S5). Me6 supplementation from 1-100 μM significantly increased the total organoid number, the budding number, and the surface area of each organoid in a dose-dependent manner (Figure 4C). The number of organoids and the budding rate in the presence of 100 μM Me6 were 1.9-fold and 1.6-fold increase than that in the presence of the control, respectively (Figure 4C). Further analysis of these organoids using immunofluorescence staining revealed that Me6 supplementation of the medium remarkably increased the percentage of Ki67^+^ cells in each organoid (Figure 4D). By employing Lgr5-EGFP-IRES-creERT2 mice, we found that the small intestine crypts cultured with Me6 showed a markedly increased percentage of Lgr5-EGFP^+^ cells on day 4 (Figure 4E). Gene expression profile analysis of these cultured organoids also showed that Me6 significantly upregulated the transcript levels of ISC-related and proliferation-promoting genes such as *Lgr5, Olfm4, Ascl2, and CyclinD1* (Figure 4F). Consistently, Me6 treatment significantly augmented the expression of Lgr5, Ascl2, and Olfm4 proteins in the organoids (Figure 4G). The intestinal organoids were then exposed to radiation and cultured in the presence or absence of Me6. Me6 supplementation markedly improved the organoid number in each well, increased the budding number and the surface area of each organoid seven days after 6 Gy radiation (Figure 4H). These observations confirm the idea that Me6 promotes normal and irradiated intestinal organoid growth and ISC proliferation *in vitro*.

### Me6 enhances intestinal organoid growth by activating β-catenin signaling

**Figure 5:**
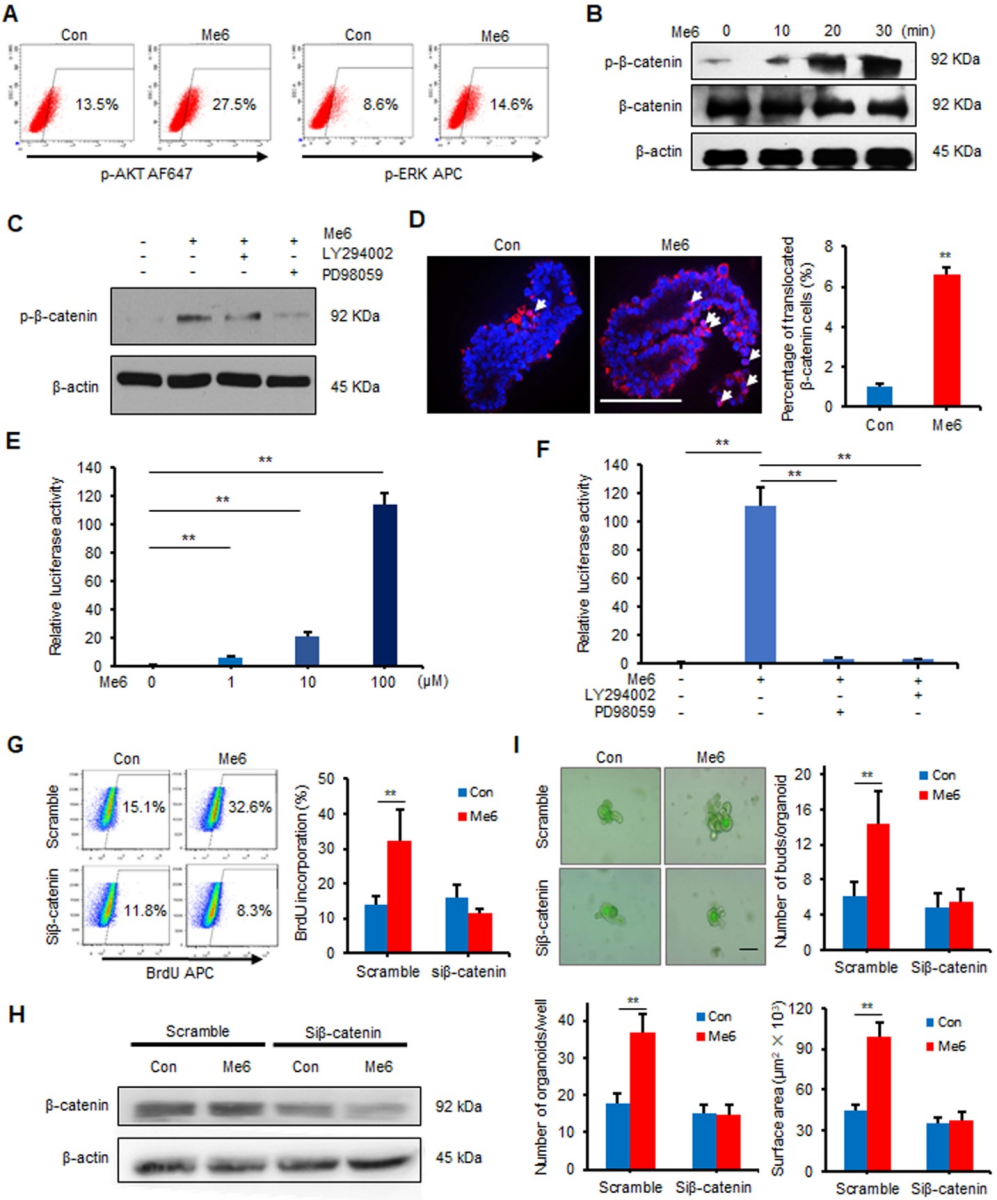
** Me6 promoted the organoid growth via activating β-catenin signaling.** (**A**) Flow cytometry analysis of ERK and PI3K pathways in IEC-6 cells treated with Me6 or PBS for 20 min. (**B**) Western blotting for the phosphorylation of β-catenin at Ser552. IEC-6 cells were stimulated with 100 µM Me6 for 0, 10, 20 or 30 min. The cell lysates were prepared and subjected to western blot analysis. (**C**)Western blot analysis of p-β-catenin in IEC-6 cells pretreated with 10 µM LY294002 or 10 µM PD98059 for 1 h and then treated with Me6 or PBS for 20 min. (**D**) Immunostaining analysis for β-catenin protein translocation in organoids treated with or without Me6 (**p < 0.01, Scale bar = 100 µm). (**E**) Relative luciferase activity of TCF-dependent reporter in IEC-6 cells treated with different concentrations of Me6 (**p < 0.01). IEC-6 cells were transfected with TCF/LEF luciferase reporter construct and were treated with Me6 for 48 h. (**F**) TCF-dependent luciferase reporter activity in IEC-6 cells pretreated with LY294002 or PD98059 for 1 h and then treated with Me6 or PBS for 48 h (**p < 0.01). (**G**) Representative dot plots and percentages of 12 h BrdU incorporation in IEC-6 cells after β-catenin or scramble siRNA transfection with or without Me6 treatment for 48 h. (**H**) Western blotting for the expression of β-catenin in the cultured small intestinal organoids in the absence or presence of Me6 with scramble or β-catenin siRNA transfection. (**I**) Representative phase contrast microscopic images of intestinal organoids cultured with or without Me6 after treated with scramble or β-catenin siRNA, and the quantification of the organoid numbers per well, the bud numbers and the surface areas per organoid (**p < 0.01, Scale bar = 100 µm).

To examine the signaling pathways for Me6 that regulate ISC proliferation, Me6-treated and vehicle-treated intestinal organoids cultured for seven days were collected and subjected to microarray analysis. Kyoto Encyclopedia of Genes and Genomes (KEGG) pathway analysis data indicated that Me6 stimulation activated several important signaling pathways including the MAPK, AKT, and β-catenin signaling pathways (Figure S6A-B). We first proved that Me6 remarkably stimulated the phosphorylation of AKT and ERK in IEC-6 cells (Figure 5A). The phosphorylation levels of β-catenin (Ser552) significantly increased in Me6-treated IEC-6 cells (Figure 5B). Notably, the addition of the PI3K/AKT or ERK inhibitors significantly suppressed the phosphorylation (Ser552) of β-catenin in Me6-treated IEC-6 cells (Figure 5C). Consistently, silencing of ERK or AKT using siRNA against *Erk1/2* or *Akt* blocked the phosphorylation of β-catenin stimulated by Me6 in IEC-6 cells (Figure S7A-C), indicating that β-catenin was a downstream target of the Me6-activated PI3K/AKT and ERK pathway. We also found that Me6 treatment significantly stimulated the phosphorylation of β-catenin and enhanced the nuclear translocation of β-catenin in small intestinal organoids (Figure S7D, Figure 5D). To confirm whether Me6 regulated the transcription of β-catenin target genes, we performed a β-catenin/TCF-dependent reporter gene assay using the TOP and FOP reporter plasmids. Me6 stimulation resulted in a dramatic increase in reporter activity in a dose-dependent manner (Figure 5E), indicating the positive transcriptional regulation of Me6 on β-catenin target gene expression. Remarkably, this upregulation of β-catenin/TCF-dependent reporter activity by Me6 was inhibited by the addition of LY294002 (an inhibitor of AKT) or PD98059 (an inhibitor of ERK) (Figure 5F). Consistently, Me6 did not increase TCF-dependent luciferase reporter activity in IEC-6 cells transfected with siRNA against the *Erk1/2* or *Akt* gene (Figure S7E). To further examine whether the effects of Me6 on crypt cell proliferation is mainly dependent on β-catenin signaling, an RNA interference technique was used to knock down the expression of β-catenin (Figure S7F-G). We then used a 12-h BrdU incorporation assay to assess the percentage of proliferating IEC-6 cells with β-catenin siRNA (siβ-catenin) transfection. Flow cytometry analysis of the percentage of BrdU^+^ cells demonstrated that the silencing of β-catenin notably inhibited Me6-induced cell proliferation (Figure 5G). Transfection of siβ-catenin in the small intestinal organoids resulted in the inhibition of β-catenin expression compared to the organoids transfected with scramble siRNA (Figure 5H). We found that Me6 supplementation in the culture medium did not significantly improve intestinal organoid number, budding number, or organoid surface area at day 7 after β-catenin knockdown (Figure 5I). Therefore, these results indicate that Me6 promotes the growth of intestinal organoids and new crypt formation mainly by activating β-catenin signaling.

### *In vivo* knockdown of β-catenin abolishes the role of Me6 in crypt regeneration in irradiated mice

**Figure 6:**
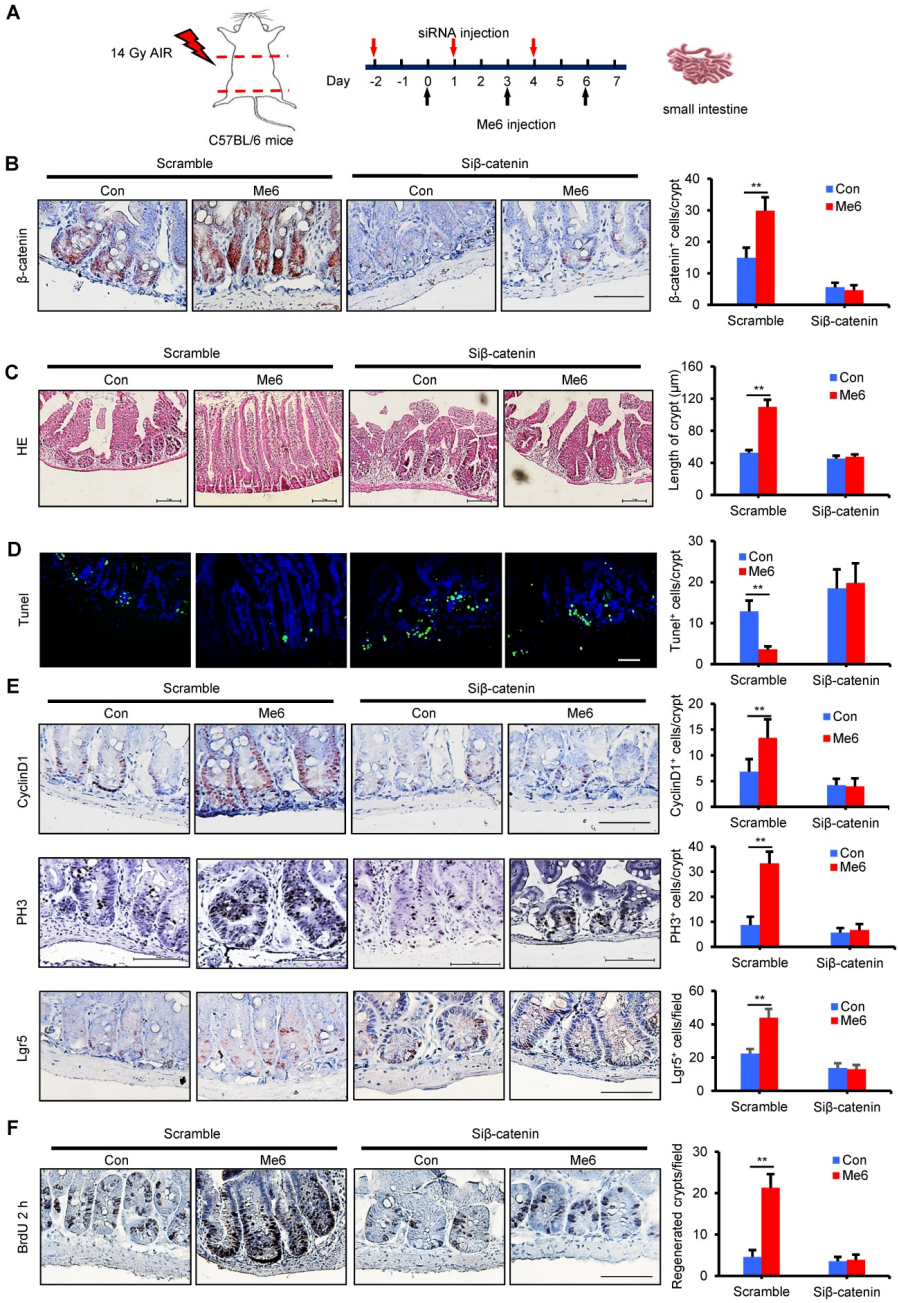
***In vivo* knockdown of β-catenin abolished the role of Me6 on crypt regeneration from irradiated mice.** (**A**) Scheme of the time schedule for scramble control siRNA (scramble)/siβ-catenin and Me6/PBS injection in mice after 14 Gy abdominal irradiation. (**B**) Analysis of the expression of β-catenin in the crypts at day 7 from irradiated mice with injection of scramble or β-catenin siRNA. Each group was treated with or without Me6 (**p < 0.01, Scale bar = 100 µm). Three mice were used in each group. (**C**) Representative HE-stained sections and the quantification of the crypt length at day 7 after irradiation (**p < 0.01, Scale bar = 100 µm). Three mice were used in each group. (**D**) Representative Tunel-stained sections and the quantification of the Tunel+ cells in the crypt of small intestine at day 7 after 14 Gy irradiation and different treatments (**p < 0.01, Scale bar = 100 µm). Three mice were used in each group. (**E**) Representative CyclinD1/PH3/Lgr5-stained sections and their quantification of them in the crypt of small intestine at day 7 after 14 Gy irradiation and different treatments (**p < 0.01, Scale bar = 100 µm). Three mice were used in each group. (**F**) The number of regenerated crypts after 14 Gy AIR, which was indicated by BrdU incorporation for 2 h (**p < 0.01, Scale bar = 100 µm). Three mice were used in each group.

**Figure 7:**
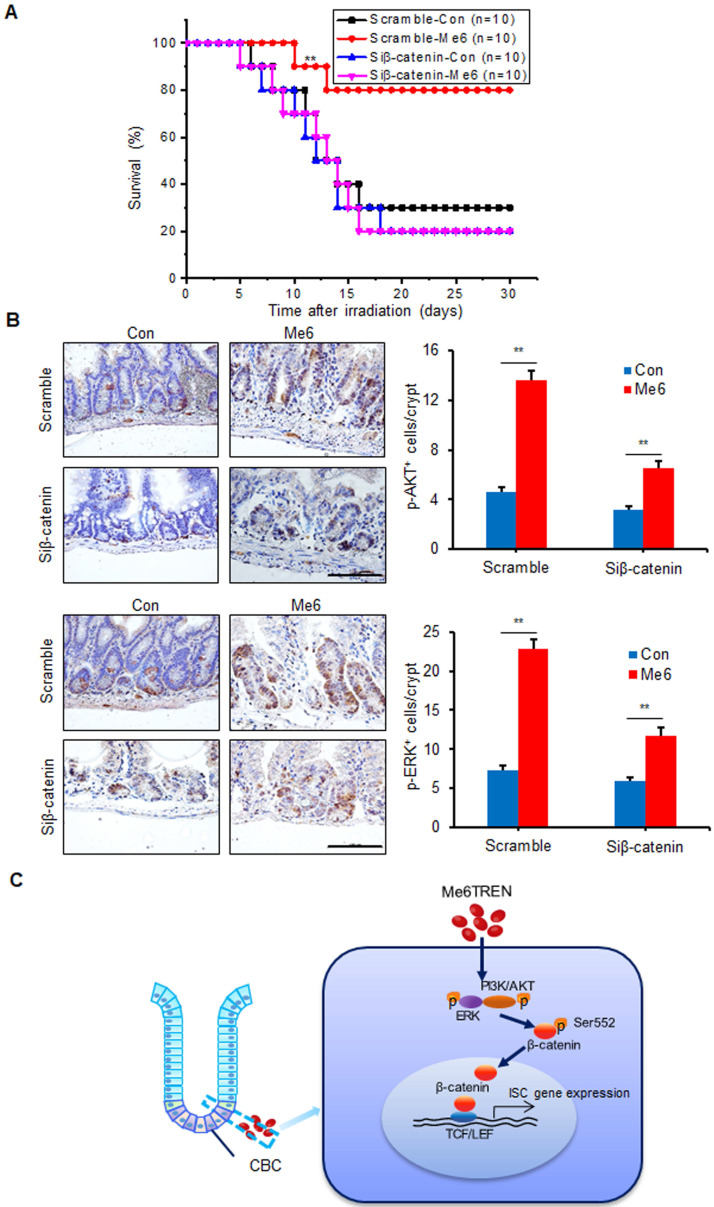
** Me6 played a beneficial role in the survival of irradiated mice mainly via activating β-catenin signals.** (**A**) Survival curves of mice after 14 Gy abdominal irradiation with control/β-catenin siRNA and Me6/PBS injection. The time schedule for these treatments is shown in Figure 6A. The number in parentheses indicates the number of animals per group. (**B**) Representative p-AKT, p-ERK-stained images and the quantification of the positive cells in the small intestine sections at 96 h after 14 Gy WBI and different treatments (**p < 0.01; Scale bar = 100 µm). Three mice were used in each group. (**C**) Graphic representation of the regulation of Me6 on ISC proliferation.

To assess whether Me6 promoted crypt regeneration via β-catenin activation* in vivo*, we injected either scramble control siRNA or siβ-catenin into C57BL/6 mice with or without Me6 administration at the indicated time points (Figure 6A). Seven days after 14 Gy AIR, the small intestines were harvested for histological evaluation. The immunohistochemical detection results for intestinal sections revealed that Me6 administration significantly increased the nuclear β-catenin staining of the crypts with scramble siRNA injection compared to the scramble siRNA control group treated with PBS (Figure 6B). Administration of siβ-catenin led to significant suppression of β-catenin expression in the crypts of mice treated with Me6 or PBS. Silencing of β-catenin abolished the effect of Me6 on enhancing the localization of nuclear β-catenin in the crypts (Figure 6B). Consistently, Me6 showed no beneficial role in the improvement of the intestinal architecture, increase in crypt length, and decrease in apoptotic cell number when the expression of β-catenin was silenced (Figure 6C-D). The injection of siRNA against β-catenin led to no significant differences in the proliferation of crypt cells that stained positively for CyclinD1 or phosphorylated histone H3 (PH3, a mitotic marker) between the Me6- and PBS-treated groups (Figure 6E). Similarly, the suppression of β-catenin expression blocked the effect of Me6 on the increase of Lgr5^+^ ISCs and regenerated crypt number after 14 Gy AIR (Figure 6E-F). The survival rate of mice subjected to 14 Gy AIR and siβ-catenin injection was independent of Me6 treatment. β-catenin knockdown impaired the effect of Me6 on the survival of mice subjected to 14 Gy AIR (Figure 7A). Notably, silencing of β-catenin could not block the phosphorylation of ERK or AKT signaling stimulated by Me6, supporting the idea that β-catenin is the downstream target of Me6-activated ERK and AKT signaling pathways (Figure 7B). Taken together, these data indicate that Me6 stimulates intestinal crypt regeneration and ISC proliferation mainly through activation of β-catenin via the ERK and AKT signaling pathways (Figure 7C).

## Discussion

In this study, we uncovered a novel role of the chemical compound Me6 in promoting ISC proliferation and crypt reproduction, thus significantly improving the survival of mice exposed to high doses of radiation. To our knowledge, only a few studies have reported on small molecules that mitigate AGS by influencing stem cell proliferation. Using an intestinal crypt cell line and an *ex vivo* enteroid model, we further showed the effect of Me6 on crypt formation and regeneration by enhancing β-catenin activity.

Intestinal crypt regeneration is required for efficient reconstitution of the normal crypt-villus structure after radiation injury. Previously, many efforts have been made to improve the regeneration of injured gut epithelia. Some growth factors, including EGF, bFGF, thrombomodulin (Thbd)-activated protein C, and MFG-E8, have shown certain effects in protecting injured intestinal tissue in animal models receiving high doses of radiation 22-27. Compared to cytokines or peptides, small molecules have several advantages regarding mitigation of gut injury, including easier storage and transportation, lower synthesis and manufacturing costs, lower immunogenicity, and improved bioavailability; thus, they are more accessible for clinical applications. One small molecule, PD0332991, an FDA-approved selective CDK4/6 inhibitor, has been reported to protect against radiation-induced intestinal injury in mice 5; however, the clinical therapeutic effect of this agent for AGS remains to be validated. The development of new small molecules that can directly enhance ISC proliferation, crypt regeneration, and accelerate intestinal recovery from radiation-induced injury is still urgently needed.

Here, we demonstrated that the small molecule Me6 showed a potent effect as a radiation countermeasure, evidenced by the fact that subcutaneous injection of Me6 into mice after lethal doses of radiation significantly increased the length of their intestinal crypt and villus by promoting intestinal epithelial cell proliferation. Of note, two doses of Me6 treatment in normal mice showed no effect on crypt and villus length and proliferation-related gene expression (Figure S8). The degree of damage of intestinal crypts post-irradiation was associated with the survival of animals 26. Notably, Me6 administration improved the survival rate of mice after 8 Gy and even 14 Gy WBI by 65% in the latter condition. These results indicated that Me6 might have a beneficial role in ISC proliferation. Activation of ISCs is known to occur within several hours after irradiation, a critical step in crypt regeneration 27, 28. Thus, we wanted to determine whether Me6 directly regulated ISCs. Using an *in vitro* clonogenic assay, we demonstrated that Me6 addition remarkably increased the number of colonies in the IEC-6 cell line after irradiation, suggesting a potential role of Me6 in crypt cell proliferation. In recent years, there have been breakthroughs in the culture of intestinal organoids 29-33. Intestinal organoids that reproduce the physiology and epithelial architecture of the intestine are more effective and useful models for the investigation of factors or regulatory mechanisms related to ISC self-renewal, proliferation, and differentiation 34. By employing a small intestinal organoid culture system from mice, we found that Me6 treatment significantly increased the budding rate of the organoids, most likely due to increased proliferation of Lgr5^+^ stem cells. Me6 supplementation also enhanced the formation of human colon organoids (Figure S9). These data provide important evidence for a novel role of Me6 in the promotion of ISC expansion and crypt formation *in vitro*. Given that the growth factors used in the intestinal organoid culture condition are essential, but not adequate, Me6 might be developed as an efficient culture supplement to sustain the self-renewal of ISCs.

To uncover the underlying mechanism of Me6 on ISC proliferation and crypt growth, we employed a microarray technique to identify potential pathways activated by Me6. KEGG pathway analysis revealed that the PI3K/AKT and ERK pathways were activated by Me6, and we further confirmed that these pathways were also activated by Me6 in IEC-6 cells. Furthermore, *in vivo* administration of the PI3K/AKT inhibitor LY294002 or ERK inhibitor PD98059 abolished the effect of Me6 on the increase in intestinal crypt and villus length (Figure S10). The microarray analysis results also suggested that β-catenin signaling and its target genes were activated. Several studies have suggested that p-AKT and p-ERK can induce the phosphorylation of β-catenin and enhance β-catenin activity 35-38. Indeed, our data showed that LY294002 and PD98059 significantly suppressed Me6-stimulated phosphorylation of β-catenin, supporting the conclusion that β-catenin was a downstream target of the Me6-activated PI3K/AKT and ERK pathways. We indeed showed that Me6 could induce TOP/FOP reporter activity, which was remarkably suppressed by LY294002 or PD98059. The role of β-catenin in promoting ISC self-renewal was mainly through stimulating target genes, including *Lgr5, Ascl2,* and* Sox9* 8, 39, 40. We revealed that Me6 was able to upregulate expression of these ISC-related and proliferation-promoting target genes. On the other hand, we verified that Me6-enhanced ISC proliferation and crypt regeneration were markedly impaired by β-catenin silencing *in vitro* and *in vivo*, confirming that β-catenin signaling is an important downstream target of Me6 in ISCs.

Recently, Jonart and their colleagues found that disruption of the central nervous system leukemia niche with Me6 did not result in an increased leukemia burden in brain parenchyma, liver, kidney, bone marrow and spleen, indicating no significant effect caused by Me6 itself on leukemia cell proliferation 41. We also evaluated the effect of Me6 on tumor cell growth after irradiation. We found that Me6 had no obvious proliferation-enhancing effect on these tumor cell lines: HCT116, LM3, and HepG2, after receiving 10 Gy irradiation (Figure S11A). To test subcutaneous Me6 administration on *in vivo* tumor growth, the tumor-bearing mice received Me6 or PBS treatment of 5 doses within 12 days after AIR at 8 Gy. There was no obvious difference in the tumor volume and weight between the Me6 and PBS treatment groups within 20 days after irradiation (Figure S11B-E). Me6 administration also had no effect on the body weight of these mice after exposure to radiation (Figure S11F). These results indicated that Me6 could not promote tumor cell growth after irradiation and showed its clinical translation potential in mitigating the acute effects of AGS via activation of the ISC regeneration process.

Taken together, our data demonstrated a novel role for Me6 in improving the survival of irradiated mice and enhancing intestinal tissue regeneration after irradiation damage. Me6 treatment significantly upregulated the expression of ISC-related and proliferation-promoting genes and proteins, and increased intestinal stem cell proliferation and organoid growth. Mechanistically, one of the main roles of Me6 in intestinal crypt regeneration is by promoting the activation of β-catenin signaling and increasing the expression of its target genes such as *Ascl2, Lgr5,* and* cyclinD1*. Overall, our work revealed that Me6 has the potential to be further developed for use in clinics to treat patients with AGS by reducing the risk of death and promoting intestinal regeneration.

## Abbreviations

Me6: Tris[2-(dimethylamino)ethyl]amine, Me6TREN
AGS: acute gastrointestinal syndrome
ISC: intestinal stem cell
CBC: crypt-based columnar cell
WBI: whole-body irradiation
AIR: abdominal irradiation
KEGG: Kyoto encyclopedia of genes and genomes
qPCR: quantitative polymerase chain reaction
HE: hematoxylin and eosin
BrdU: bromodeoxyuridine

## Supplementary Material

Supplementary figures and tables.

Supplementary video S1-Control.

Supplementary video S1-Me6.
