# Supplementary material for: Me6TREN targets β-catenin signaling to stimulate intestinal stem cell regeneration after radiation
Source: Theranostics. 2020 Aug 13;10(22):10171–85. doi: 10.7150/thno.46415 (PMC7481405; doi:10.7150/thno.46415)
Supplement: Supplementary file 1 — Supplementary figures and tables. [file thnov10p10171s1.pdf]

Supplementary Materials and Methods

Mice and radiation

Animals were housed under standardized conditions with controlled temperature and humidity and a 12/12-h day/night light cycle. C57BL/6 were obtained from commercial vendor (Beijing Vital River Laboratory Animal Technology Co. Ltd). Lgr5-EGFP-IRES-creERT2 mice were a kind gift from Professor Yuwen Cong (Beijing Institute of Radiation Medicine, Beijing, China). Animal procedures and protocols were conducted in accordance with the institutional review board of the Academy of Military Medical Sciences.

Me6 treatment

To assess the best dose of Me6 on the survival of mice after 8 Gy radiation, we administered the small molecule compound Me6 (Sigma) to the mice at doses of 0, 2.5, 5, or 10 mg/kg. To determine the optimal schedule of Me6, Me6 was injected to mice daily, every other day, every two days or every three days until 12 days after radiation exposure. Control animals were injected with the same volume of PBS. For the normal mice, we administered with Me6 at 2.5 mg/kg or PBS on day 0 and day 3. Then we sacrificed mice at 96 h after the first administration and isolated the intestinal crypts for HE staining and qPCR analysis.

TUNEL staining

A terminal deoxynucleotidyl transferase dUTP nick end labeling (TUNEL) assay was performed on intestinal slides according to the manufacturer's instructions (Promega). Images were captured using a Nikon fluorescence microscope. Quantitation of the TUNEL assay was done by counting the total number of DAPI stained cells (blue) and total number of the TUNEL positive cells (green) to calculate a ratio, with 3 mice per group, and at least 30 villi or crypts per mouse were used for counting the positive cell number. The apoptotic index was calculated as the proportion of $DAPI^+$ (blue) cells that stained for TUNEL (green).

Microarray assays

C57BL/6 mice were given 2.5 mg/kg Me6 or PBS on day 0 and day 3 after 14 Gy WBI. Four days after irradiation, the jejunum were collected and the crypts were harvested for microarray detection by an Illumina profiling chip. Samples from three independent experiments were detected and analyzed. Fresh isolated intestinal crypts were cultured in the standard organoid medium in the presence or absence of 100 μM Me6. Four days later, RNA of these organoids was purified for microarray detection by an Agilent profiling chip.

Human colon organoid culture

The human intestinal segment was obtained from adjacent normal colon tissues of colon cancer removed from patients. The colon tissue was washed with ice-cold Dulbecco’s Phosphate buffered saline (DPBS) and the stroma was removed using fine scissors. Then the remaining epithelium was cutted into 1 mm^3

pieces. The tissue fragments were washed 3-4 times with cold DPBS and were incubated in 2.5 mM EDTA at 4 ℃ for 1 h on ice with gentle rocking to release crypts. Liberated crypts were filterd twice through a 150 μm filter and approximately 200 isolated crypts were embedded in Matrigel and cultured in crypt culture medium (IntestiCult™ Organoid Growth Medium, STEM CELL, 06010). The medium was changed every 2 days.

Colony-forming Assays

IEC-6 cells were given a total dose of 10 Gy radiation with a ^{60}Co source. A total of 5000 irradiated cells were cultured in a 6-well plate with or without 100 μM Me6 for 7 days. Colonies were stained with crystal violet and then counted.

Reverse transcription–polymerase chain reaction

Total RNA was isolated using TRIzol Reagent (Invitrogen) according to the manufacturer's protocol, and 500 ng of total RNA was reverse-transcribed into cDNA using M-MLV reverse transcriptase (TaKaRa) in a 10 μL reaction mixture. Real-time quantitative PCR (qPCR) analysis was performed on a Bio-Rad iQ5 system using the SYBR Green PCR Master Mix (TaKaRa), and all the experiments were repeated 3 times. The primers were described in detail in the Supplemental Table 1.

5-bromo-2-deoxyuridine (BrdU) incorporation assays

To evaluate the BrdU incorporation rate in crypt cells, we injected mice with BrdU (100 mg/kg; Sigma Chemical Co., St. Louis, MO) 16 h prior to sacrifice. Immediately after the animal was sacrificed, the middle part of jejunum was collected and carefully rinsed with ice-cold physiological saline, then fixed with 4% paraformaldehyde for at least 24 h before it was embedded in paraffin. For BrdU staining, intestinal sections were deparaffinized and treated with 2 M HCl for 20 min. The staining was carried out following a standard protocol with an anti-BrdU antibody (CST, 5292), secondary antibody and developed with NovaRED. BrdU-positive cells were counted in 40× fields, and the percentage of the BrdU-positive cells in the intestinal crypt was determined by counting 30 or more crypts and reported as the mean ± SD. To enumerate crypt cell regeneration, crypts were scored 4 days after irradiation in PBS- or Me6-treated mice by injecting 100 mg/kg of BrdU (Sigma-Aldrich, St. Louis, MO) 2 h prior to the harvest of the jejunum, and immunohistochemistry was performed as above-mentioned. For the definition of BrdU labeled regenerated crypts, it contained 10 or more BrdU-positive cells. Three mice were used in each group.

In Situ Hybridization

The jejunum was fixed in 4% paraformaldehyde for 24 h and then embedded with paraffin. The intestinal sections were prepared and treated using RNAscope In Situ Hybridization 2.5 HD red assay kit (Advanced Cell Diagnostic, Newark, CA). In brief, the tissue underwent target retrieval, permeabilization, hybridization of Lgr5 (Hs-Lgr5, 311021) and WDR43 (Hs-WDR43, 472711), amplification, and visualization using DAB-A and DAB-B. Sections were imaged using a Nikon microscope.

Western blot analysis

For signal pathway detection, IEC-6 cells were treated with Me6 (100 μM) for different time points. For the ISC key protein assays, fresh intestinal crypts were cultured in standard organoid medium in the presence or absence of Me6 (100 μM). Cells were collected and lysed directly in lysis buffer. Cells were lysed in radioimmunoprecipitation assay (RIPA) buffer containing a complete protease inhibitor cocktail tablet. Protein samples were separated on 10% polyacrylamide gels and transferred to polyvinylidene difluoride membranes. The membranes were incubated with primary antibodies overnight at 4 ℃, and detection was performed with peroxidase-conjugated goat anti-rabbit or anti-mouse IgG using a chemiluminescence detection system. The antibodies used were as follows: anti-Lgr5 (R&D, MAB82), anti-β-catenin (CST, 8480S), anti-p-β-catenin (CST, 5651S), anti-Olfm4 (Abcam, ab850460), anti-Ascl2 (Abcam, ab176334), anti-β-actin (CST, 12262S), anti-AKT (CST, 9272), anti-ERK1/2 (CST, 4695), anti-p-AKT (4060), anti-p-ERK1/2 (CST, 4370).

Flow cytometry assay

Cell samples were trypsinized into single-cell suspensions using 0.25% trypsin (Gibco) and resuspended in PBS containing 5% FBS. The cells were washed and incubated with isotypic antibodies or the indicated antibodies in the dark for 45 min at 4 ℃. After incubation, the cells were washed three times with PBS and suspended in 0.4 mL of PBS for analysis. 7-Aminoactinomycin D (BD Biosciences) was added to each sample and incubated for 5 min before analysis to eliminate the dead cells. Flow-cytometry analysis was performed using FACSCalibur (BD Biosciences). The antibodies used were as follows: anti-p-AKT (CST, 4060S); anti-p-ERK (CST, 4367S); anti-BrdU (CST, 5292S).

Fluorescein isothiocyanate (FITC)-Dextran test

C57BL/6 mice were administered 2.5 mg/kg Me6 or PBS on day 0 and day 3 after 14 Gy WBI. Four days after irradiation, FITC-Dextran (Sigma-Aldrich, St. Louis, MO) was administered to the mice by oral gavage at a concentration of 0.6 mg/g body weight and a volume of 200 μL. Four hours after gavage, the serum of mice were collected, and 50 μL of both diluted serum samples and standards as well as blanks (PBS and diluted serum from untreated animals), were transferred to black 96-well microplates. FITC-Dextran concentrations were analyzed with a fluorescence spectrophotometer and fluorescence intensity was measured (excitation, 492 nm; emission, 525 nm).

EdU incorporation and detection

HepG2, LM3, and HCT116 cells were cultured in Dulbecco's Modified Eagle Medium (DMEM) supplemented with 10% fetal bovine serum (FBS). EdU (10 μM) was added to the culture medium for 2 h before the cells were trypsinized into single-cell suspensions using 0.25% trypsin and suspended in PBS containing 1% bovine serum albumin (BSA). The pellet was collected, 100 μL of Click-iT™ fixative was added, mixed well, and incubated for 15 min at room temperature. Cells were washed with 3 mL of 1% BSA in PBS, the cells were pelleted, and the supernatant was removed. The cell pellet was collected and cells were suspended in 100 μL of 1× Click-iT™ permeabilization and wash reagent. The

cells were incubated for 15 min and then subjected to detection. Five hundred microliters of Click-iT™ Plus reaction cocktail was added to each tube of fixed cells, and the reaction mixture was incubated for 30 min at room temperature. Cells were washed once with 3 mL of 1× Click-iT™ permeabilization and the cells were analyzed using flow cytometry.

Tumor inoculation and detection

Ten million HCT116 cells suspended in 100 μL PBS were injected into the subcutaneous tissue of the flank region of BALB/c nude mice. Two weeks after tumor cell inoculation, tumor-bearing mice received abdominal X-ray irradiation at a dose of 8 Gy, and were injected with PBS or Me6 at 2.5 mg/kg on days 0, 3, 6, 9, and 12 after irradiation. Following irradiation, tumor volume (V) was determined by measuring the length (L) and width (W) of the tumor using vernier calipers, and calculated as $V = L \times W^2/2$ and body weight were monitored every other day for 20 days.

Supplementary Figures, Tables and Video

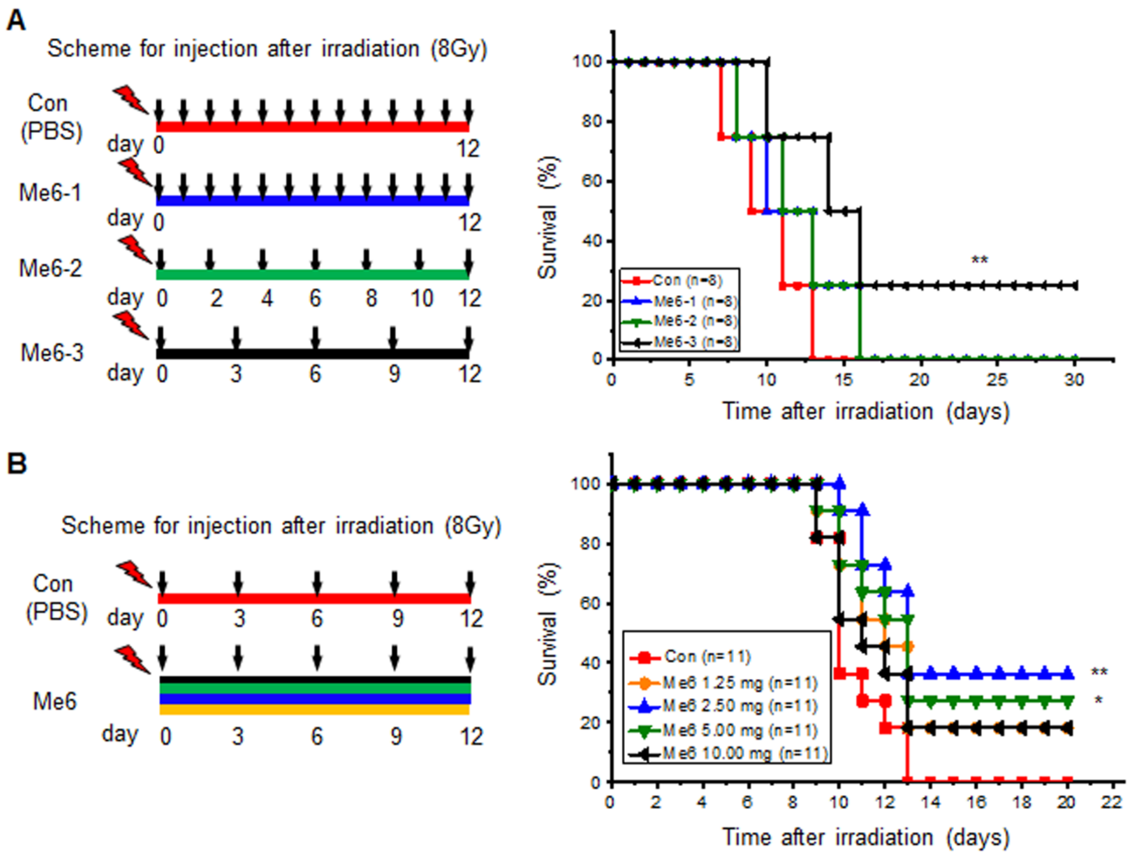

Figure S1. Me6 treatment increased the survival rate of irradiated C57BL/6 mice.

(A) Survival curves of mice after 8 Gy whole body irradiation (WBI) and Control (PBS) or Me6 treatment. Me6 at 5 mg/kg or PBS were administered on the indicated days after irradiation (left). Eight mice were used in each group. (**$p < 0.01$).

(B) Survival curves of mice after 8 Gy WBI and different doses of Me6 injection. Me6 at 0, 1.25, 2.5, 5, and 10 mg/kg were administered to the mice on the indicated days after irradiation (left). Eleven mice were used in each group. (*$p < 0.05$, **$p < 0.01$)

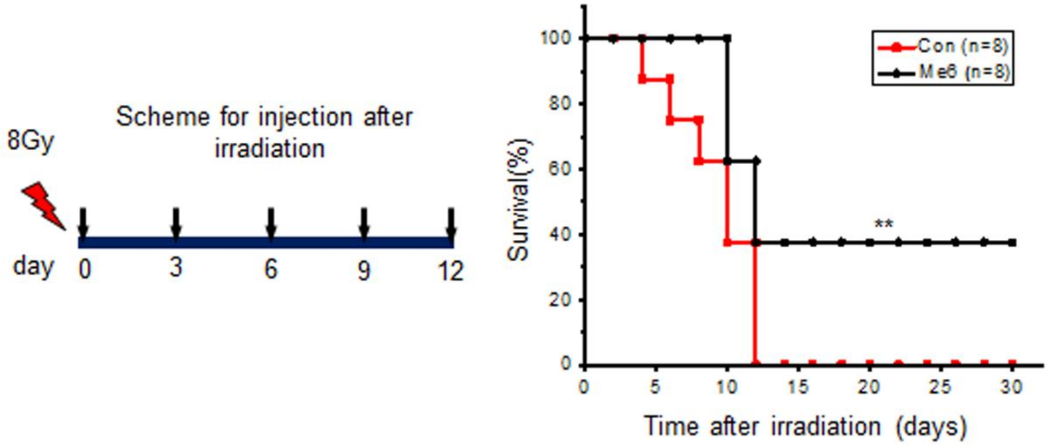

Figure S2. Me6 treatment increased the survival rate of irradiated BALB/c mice.

BALB/c mice were administered with Control (PBS) or 2.5mg/kg Me6 treatment after 8 Gy WBI. Eight mice were used in each group (**p < 0.01).

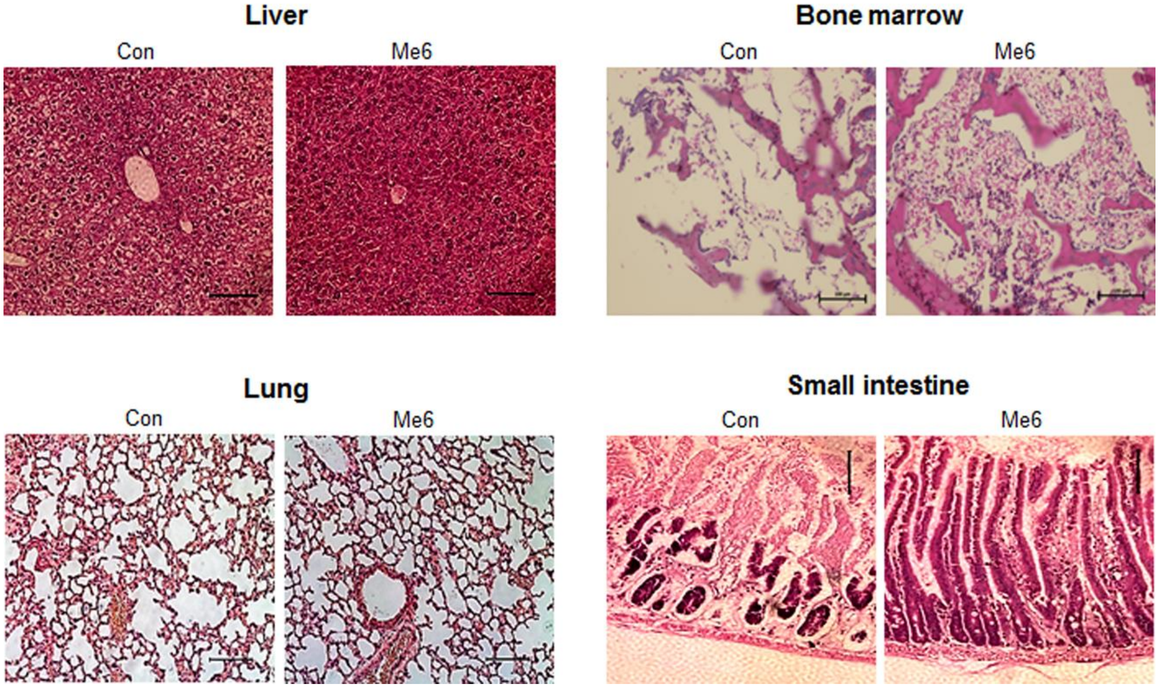

Figure S3. The repair effect of Me6 on radiation-injured tissues and organs.

Representative HE-stained sections of liver, bone marrow, lung and small intestine on day 7 after 8 Gy WBI. The irradiated mice were administered with Me6 or PBS according to the time schedule shown in Figure1B, Scale bar = 100 μm.

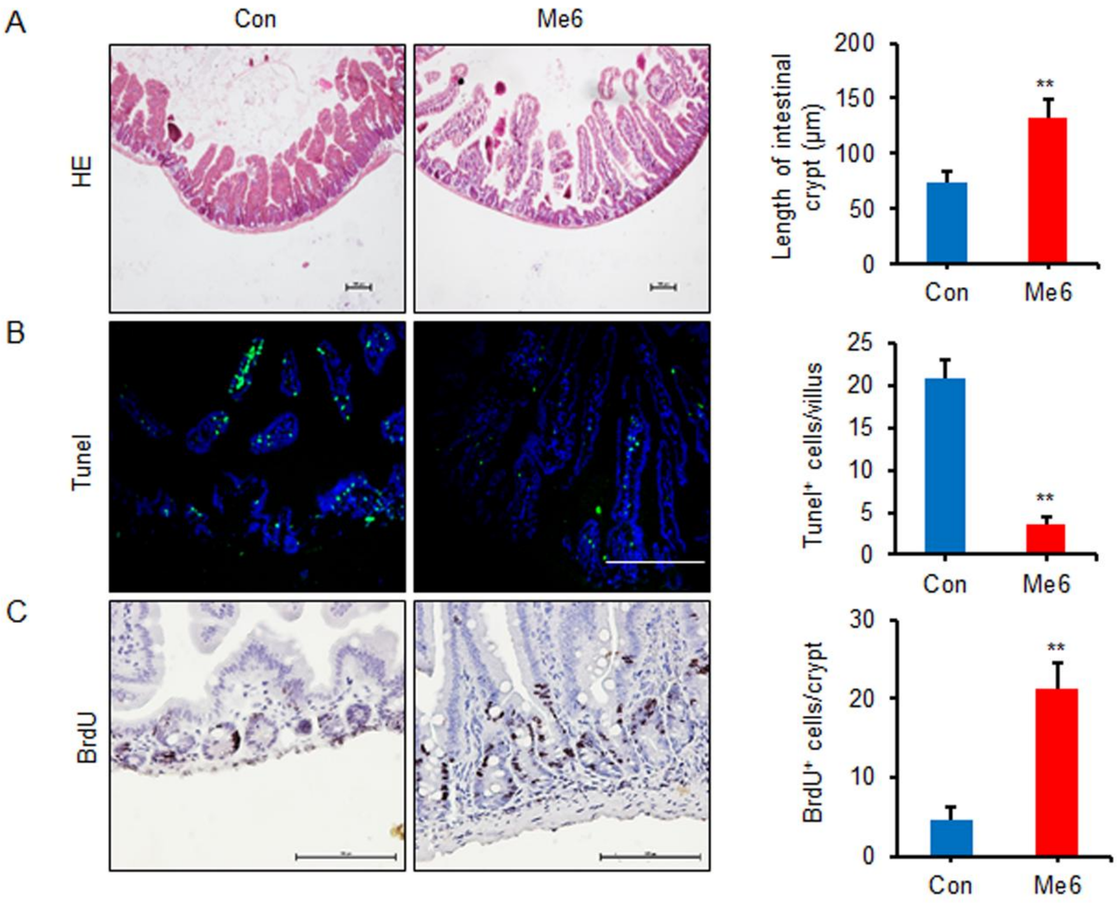

Figure S4. Me6 promoted intestinal crypt regeneration post 14 Gy abdominal irradiation.

(A) Representative HE-stained sections and the quantification of the crypt length on day 7 after 14 Gy abdominal irradiation (**p < 0.01). Mice were given 2.5 mg/kg Me6 or PBS on the indicated days after radiation. Three mice were used in each group.

(B) Representative Tunel-stained sections and the quantification of the $Tunel^+$ cells in the villus of jejunum on day 7 after 14 Gy abdominal irradiation (**p < 0.01, Scale bar = 100 μm). Three mice were used in each group.

(C) Representative BrdU-stained sections and the quantification of the $BrdU^+$ cells in the crypt on day 7 after 14 Gy abdominal irradiation (**p < 0.01, Scale bar = 100 μm). Three mice were used in each group.

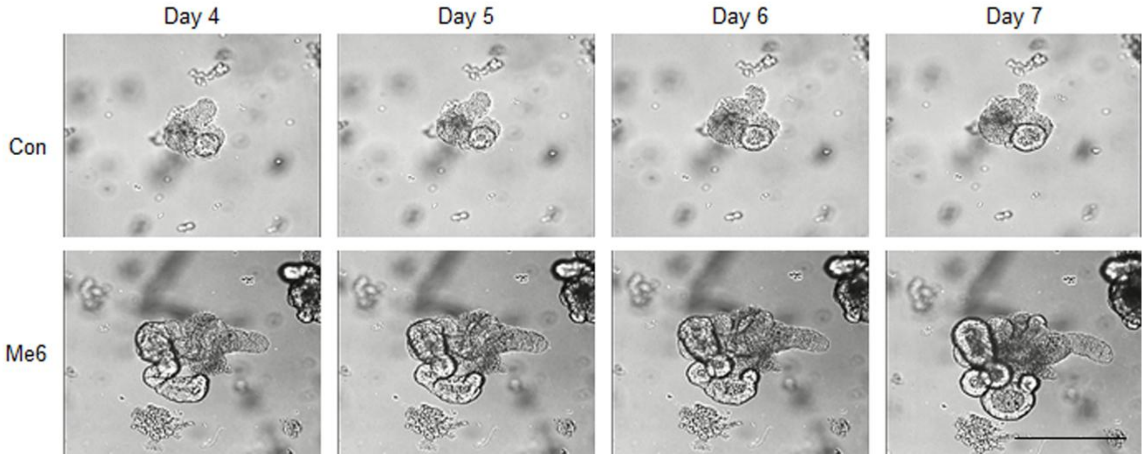

Figure S5 Time-lapse imaging of intestinal organoids with or without Me6 on day 4-7.
The small intestinal organoids of mice were cultured in the standard medium with or without Me6 and were observed using microscope from day 4 to day 7 **(**Scale bar = 100 μm).

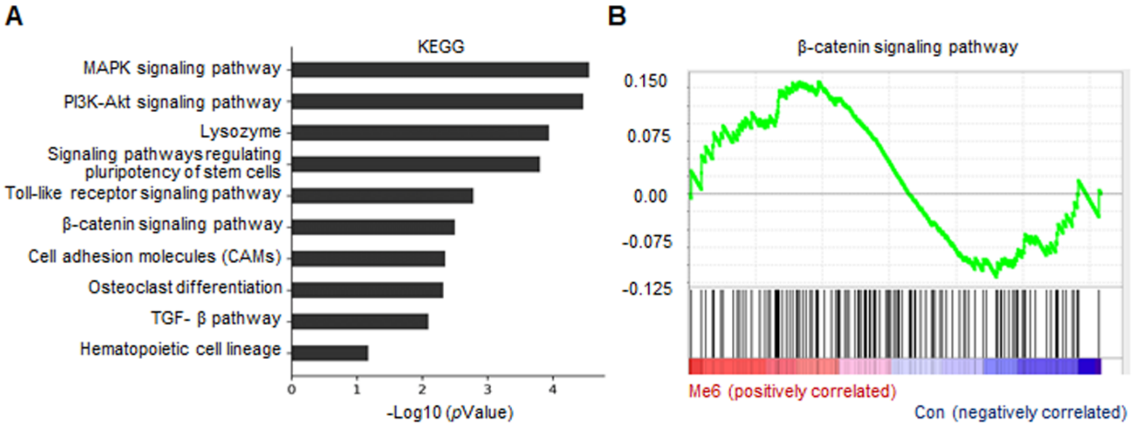

Figure S6. The activated signaling pathways by Me6 were predicted using the microarray data.
(A-B) The KEGG pathway analysis data using the cultured intestinal organoids. The intestinal organoids were cultured in standard medium with or without Me6 supplementation. At day 4, microarray assays were performed using samples prepared from these organoids.

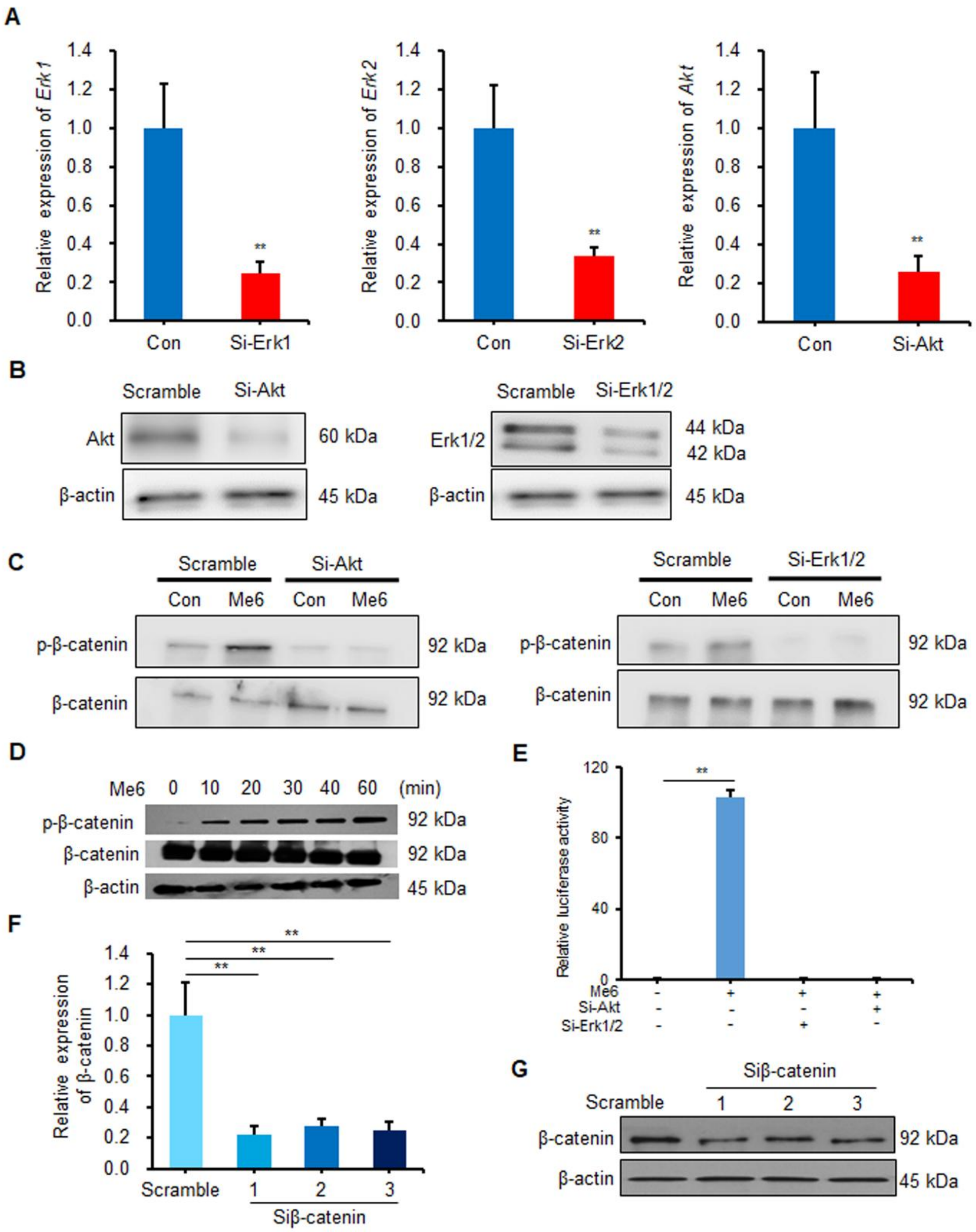

Figure S7. β-catenin was a downstream target of the Me6-activated PI3K/AKT and ERK pathway.

(A) qPCR detection for *Erk1*, *Erk2* and *Akt* gene expression in IEC-6 cells after Si-Erk1, Si-Erk2, Si-Akt or scramble siRNA transfection (**p < 0.01).

(B) Western blotting detection for ERK and AKT protein expression in IEC-6 cells after Si-Erk1/2, Si-Akt or scramble siRNA transfection.

(C) Western blotting for the expression of p-β-catenin in IEC-6 cells transfected with Si-Erk1/2, Si-Akt or scramble siRNA for 48 h and then treated with Me6 or PBS for 20 min.

(D) Western blotting for the phosphorylation of β-catenin. Organoids were stimulated with 100 μM Me6 for 0, 10, 20, 30, 40, or 60 min. The cell lysates were prepared and subjected to western blot analysis.

(E) TCF-dependent luciferase reporter activity in IEC-6 cells transfected with Si-Erk1/2, Si-Akt or scramble siRNA and treated with Me6 or PBS for 48 h (**$p < 0.01$). IEC-6 cells were co-transfected with TCF/LEF luciferase reporter construct for 48 h.

(F) qPCR detection for β-catenin gene and protein expression in IEC-6 cells after β-catenin or scramble siRNA transfection.

(G) Western blotting detection for β-catenin gene and protein expression in IEC-6 cells after β-catenin or scramble siRNA transfection.

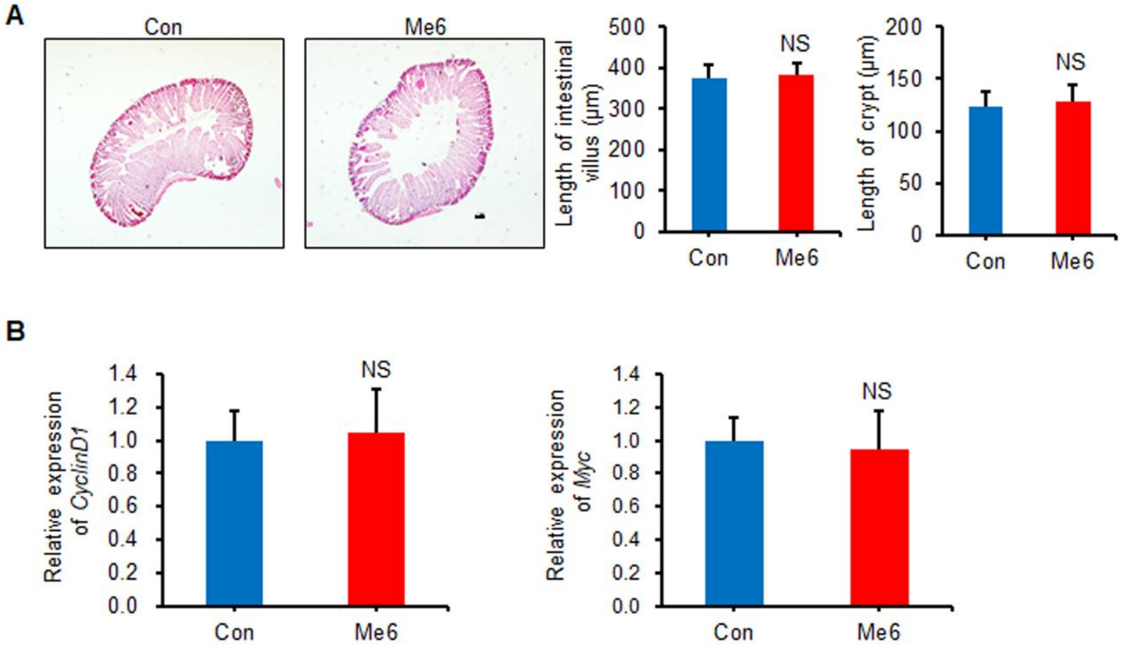

Figure S8. Me6 showed no obvious effect on the crypt and villus length and proliferation-related gene expression in the small intestinal epithelium of normal mice.

(A) Representative HE stained sections and the quantification of the villus and crypt length of normal mice administered with PBS or Me6 for two doses on day 0 and day 3 (Scale bar = 100 μm). Three mice were used in each group. Intestinal sections were prepared on day 4 after the first dose of Me6.

(B) qPCR detection for the expression of *CyclinD1* and *Myc* genes in the intestinal crypts on day 4 after administration with PBS or Me6.

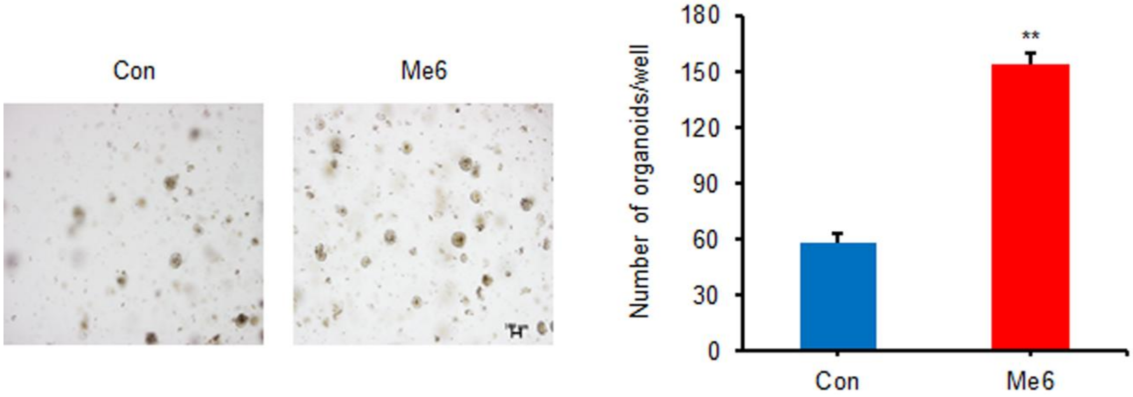

Figure S9. Me6 treatment enhanced the formation of human colon organoids.

The human colon organoids were cultured in the standard medium with or without Me6 supplementation. The morphology and the total number of the organoids were observed under a microscope on day 7 (**p < 0.01; scale bar = 100 μm**).**

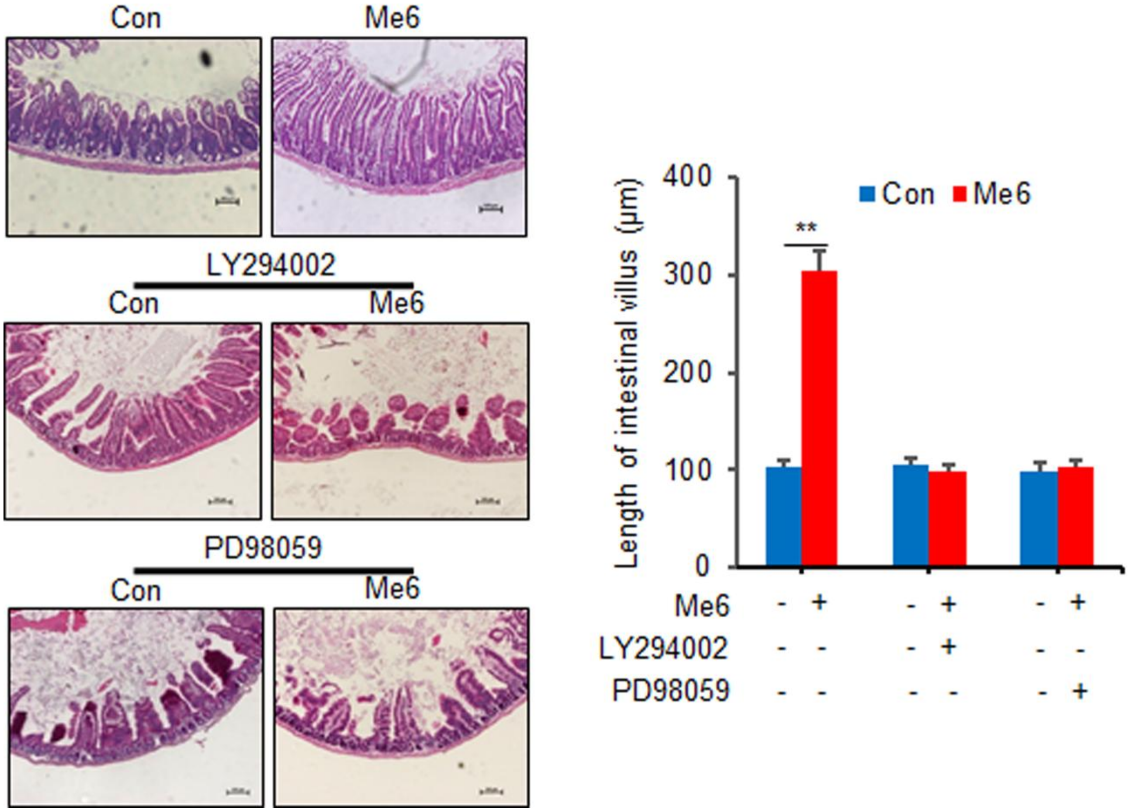

Figure S10. Representative HE–stained intestinal sections and the quantification of the villus length on day 7 after 8 Gy WBI.

Mice were injected with inhibitor, Me6 or PBS on the indicated days after irradiation (**p < 0.01, Scale bar = 100 μm). Three mice were used in each group, and at least 30 intact villi were counted and reported as the mean ± SD.

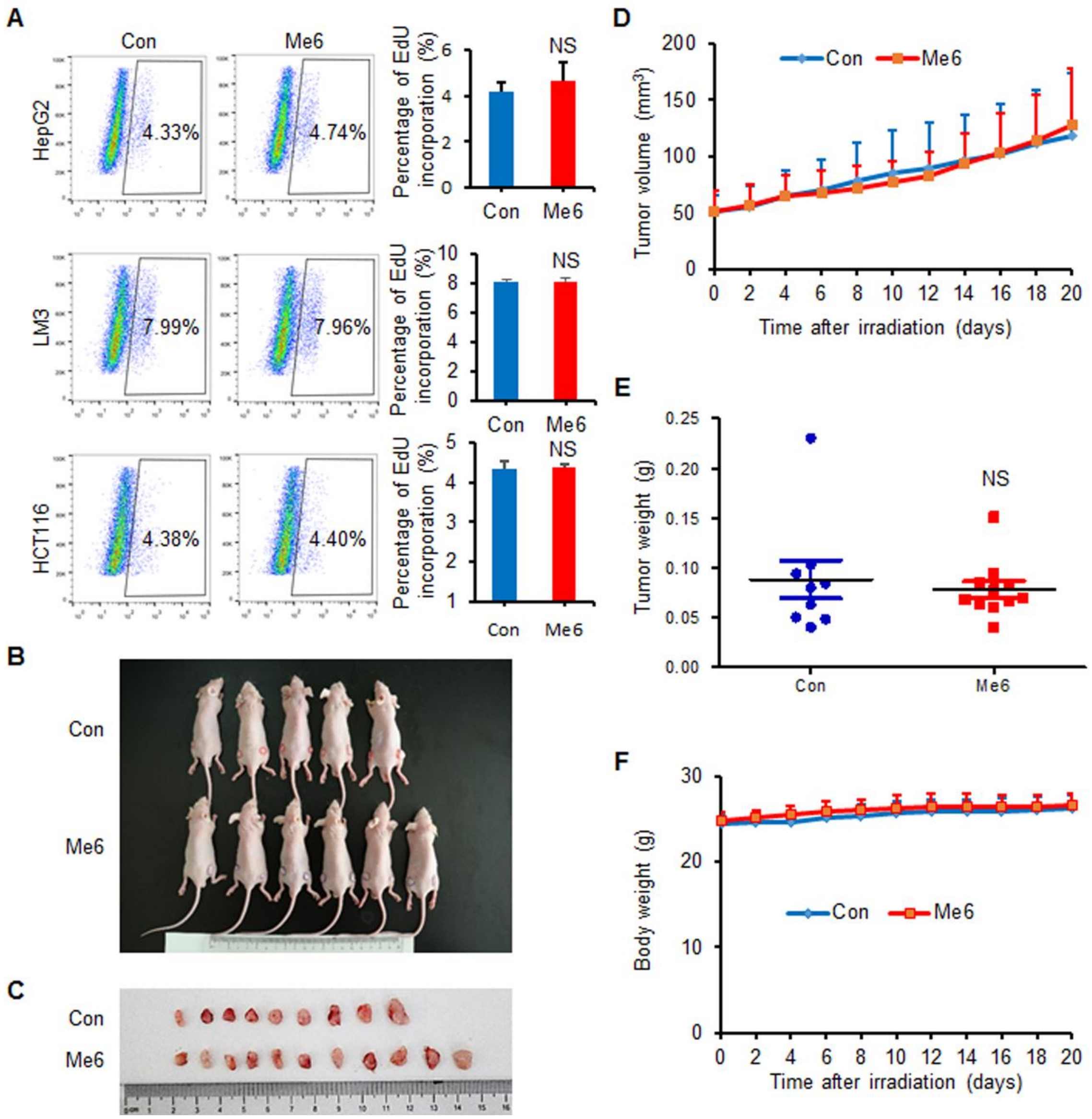

Figure S11. Me6 showed no obvious effect on tumor proliferation from radiation.

(A) The percentage analysis of EdU incorporation in HepG2, LM3 and HCT116 cells with or without the addition of Me6 (100 μM) to the culture medium for 48 h after exposure to 10 Gy radiation (NS, no significant difference).

(B-C) Gross observation of tumor-bearing mice (B) and peeled tumors from these mice (C) on day 20 after receiving AIR and different treatments.

(D) The mean tumor volume of the HCT116 cell-formed tumors in nude mice was monitored within 20 days after receiving AIR and different treatments.

(E) Tumor weight of Me6 compared to PBS groups was examined after mice were sacrificed on day 20 after radiation.

(F) The body weight of the tumor-bearing mice was monitored within 20 days after receiving AIR and different treatments.

Table S1. Table of primers used in study.

Lgr5	CCTACTCGAAGACTTACCCAGT
	GCATTGGGGTGAATGATAGCA
Bmi	AAATCCCCACTTAATGTGTGTCC
	CTTGCTGGTCTCCAAGTAACG
Ascl2	AAGCACACCTTGACTGGTACG
	AAGTGGACGTTTGCACCTTCA
Olfm4	CAGCCACTTTCCAATTTCACTG
	GCTGGACATACTCCTTCACCTTA
Mam1	GCCCGACGAAGACATGAAGG
	CTTGTGGAGAACCTAACTGCTC
Myc	CCCTATTTCATCTGCGACGAG
	GAGAAGGACGTAGCGACCG
Jun	ACTCGGACCTTCTCACGTC
	GGTCGGTGTAGTGGTGATGT
CyclinD1	CTGTGCTGCGAAGTGGAAACCAT
	TTCATGGCCAGCGGGAAGACCTC
FOS	CCAGTCAAGAGCATCAGCAA
	AAGTAGTGCAGCCCGGAGTA
Axin2	CTCCCCACCTTGAATGAAGA
	ACTGGGTCGCTTCTCTTGAA
Bcl2	ACATGCTCGCCCAGCCAC
	TGCACATGGGGCATCTTGG
β-actin	AGCTGAGAGGGAAATCGTGCG
	GTGCCACCAGACAGCACTGTG
Erk1	GCTGACCCTGAGCACGACCA
	CTGGTTCATCTGTCGGATCA
Erk2	TTGCTGAAGCACCATTCAAG
	ACGGCTCAAAGGAGTCAAGA
Akt	ACTCATTCCAGACCCACGAC
	CCGGTACACCACGTTCTTCT

Table S2. Table of siRNAs used in study.

**Si-Erk1**	GGACCGGAUGUUAACCUUUTT
	AAAGGUUAACAUCCGGUCCTT
**Si-Erk2**	GCUAGAAACUAUUUGCUUUTT
	AAAGCAAAUAGUUUCUAGCTT
**Si-Akt**	GAAUGAUGGCACCUUUAUUTT
	AAUAAAGGUGCCAUCAUUCTT

Video S1. Time-lapse imaging video of intestinal organoids.

Small intestinal organoids were cultured in standard organoid medium with or without Me6 supplementation and were observed under a time-lapse microscope from day 4 to day 7.

Video S1 - Control.mp4

Video S1 - Me6.mp4
